# *Staphylococcus aureus*-specific TIGIT^+^ Treg are present in the blood of healthy subjects – a hurdle for vaccination?

**DOI:** 10.3389/fimmu.2024.1500696

**Published:** 2025-02-04

**Authors:** Jonah Clegg, Malgorzata E. Mnich, Alberto Carignano, Giovanni Cova, Simona Tavarini, Chiara Sammicheli, Bruna Clemente, Megan Smith, Emilio Siena, Monia Bardelli, Michela Brazzoli, Fabio Bagnoli, Rachel M. McLoughlin, Elisabetta Soldaini

**Affiliations:** ^1^ GSK, Research Center, Siena, Italy; ^2^ Host Pathogen Interactions Group, School of Biochemistry and Immunology, Trinity Biomedical Sciences Institute, Trinity College Dublin, Dublin, Ireland; ^3^ Department of Medical Microbiology, University Medical Center Utrecht, Utrecht, Netherlands

**Keywords:** *Staphylococcus aureus*, Th17, Treg, TIGIT, host-pathogen interactions, vaccines, colonization

## Abstract

*Staphylococcus aureus* poses an enormous burden of morbidity and mortality worldwide. Making an efficacious vaccine has however proven extremely challenging. Due to colonizing interactions, pre-existing *S. aureus*-specific CD4^+^ T cells are often found in the human population and yet a detailed characterization of their phenotypes and how they might in turn impact vaccine efficacy are thus far unknown. Using an activation induced marker assay to sort for *S. aureus*-specific CD4^+^ T cells in an effector function-independent manner, single cell transcriptomic analysis was conducted. Remarkably, *S. aureus*-specific CD4^+^ T cells consisted not only of a broader spectrum of conventional T cells (Tcon) than previously described but also of regulatory T cells (Treg). As compared to polyclonally-activated CD4^+^ T cells, *S. aureus*-specific Tcon were enriched for the expression of the Th17-type cytokine genes *IL17A*, *IL22* and *IL26*, while higher percentages of *S. aureus*-specific Treg expressed the T Cell Immunoreceptor with Ig and ITIM domains (TIGIT), a pleiotropic immune checkpoint. Notably, the antagonistic anti-TIGIT mAb Tiragolumab increased IL-1β production in response to *S. aureus in vitro*. Therefore, these results uncover the presence of *S. aureus*-specific TIGIT^+^ Treg in the blood of healthy subjects that could blunt responses to vaccination and indicate TIGIT as a potential targetable biomarker to overcome pre-exposure-induced immunosuppression.

## Introduction

*Staphylococcus aureus* is a gram-positive bacterium capable of asymptomatic colonization as well as causing a spectrum of diseases in the human population, therefore meeting the definition for classification as a pathobiont. In 2019 *S. aureus* was the only bacterium responsible for more than one million deaths globally (1). Compounding this issue, *S. aureus* displays an alarming propensity to develop resistance against antimicrobial drugs with methicillin-resistant *S. aureus* identified as the single deadliest antimicrobial-resistant drug-pathogen combination (2). As a result, the development of novel therapeutic strategies such as a vaccine is considered an urgent and unmet public need. There have been multiple attempts in designing a vaccine against *S. aureus*, all of which have thus far ended in failure when trialed in a clinical setting (3). Numerous factors have been suggested to explain such failures including a need for better models of infection and a lack of defined correlates of protection, and yet although known as a human commensal, the relevance of colonization-induced pre-existing immune responses to *S. aureus* has been consistently overlooked in vaccine development and clinical study design.

*S. aureus* is part of the normal human microbiome, capable of colonizing all body surfaces such as the nasal cavity, skin, lungs and intestine (4–8). Strikingly, within the first 8 weeks of life 40% of newborns were found to be colonized with *S. aureus* (9). The intricacy of this human-*S. aureus* relationship was recently exemplified by the finding that *S. aureus* undergoes on-person evolution during long-term colonization in atopic dermatitis (10). As a result of such an early and frequent interaction, the vast majority of individuals have detectable pre-existing humoral and cellular immune responses to *S. aureus* (11–14), that do not prevent from the onset of infection. In addition, previous *S. aureus* infection often does not protect from re-infection, as exemplified by the high incidence of recurrencies in skin and soft tissue infection caused by *S. aureus*, showing that natural immunity is only partially efficacious (15). Pre-existing immune responses are known to significantly affect vaccine responses in humans, a phenomenon called antigenic sin or immunological imprinting, as seen during influenza and SARS-CoV-2 vaccination (16–18). In fact, research conducted in mouse models has showcased the negative implications that pre-exposure-induced immune responses may have on *S. aureus* vaccine efficacy (19–21). Whether and how pre-existing immunity to *S. aureus* hinders vaccine efficacy in humans is however as of yet, unknown.

Studies in mice have highlighted protective roles played by antibodies in various *S. aureus* infection models (21–23). However, the fact that all previous failed vaccines were designed to generate antibody-mediated-*S. aureus* immune responses and that individuals with primary or secondary B cell immunodeficiencies have no increased risk of *S. aureus* infection has emphasized the importance of anti-*S. aureus* cell mediated immune responses (24–26). Multiple murine studies have linked CD4^+^ T cells, and in particular Th17 and/or Th1 cells and their signature cytokines, IL-17 and IFN-γ, to protective immunity during *S. aureus* infections and vaccination (27–29). In humans both genetic and clinical evidence has suggested that CD4^+^ T cells and specifically Th17 are linked to protection from *S. aureus* infections at barrier sites such as the skin and lungs (30, 31). Specifically, inborn errors of immunity have shown the pivotal role of the IL-17 axis in mucocutaneous immunity to *S. aureus* in humans (32). As a result, CD4^+^ T cells have been deemed a crucial target for future *S. aureus* vaccines. Previous studies investigating pre-existing CD4^+^ T cell responses specific to *S. aureus* in healthy subjects have evidenced a Th17 and/or Th1 response based on proliferation and/or cytokines production (11, 13, 33).

Since memory CD4^+^ T cells form a continuum, shaped by the microbes and the tissue microenvironment, rather than defined Th subtypes and can adapt their functions in response to changing circumstances, a feature called plasticity (34), we aimed to capture the complexity of the human pre-existing CD4^+^ T cell response to *S. aureus*. To achieve this goal, *S. aureus*-specific CD4^+^ T cells were identified in the blood of healthy subjects using a sensitive, HLA-agnostic and effector function-independent activation induced marker (AIM) assay, and the transcriptome of single AIM^+^ cells was analyzed by Sorting and Robot-assisted Transcriptome Sequencing (SORT-seq). This method captured not only the complexity of cytokines produced by *S. aureus*-specific CD4^+^ T cells in different states, likely reflecting the continuum of Th17-type memory cells present in humans (35), but also identified *S. aureus*-specific Treg, which have been previously largely overlooked. Remarkably, the vast majority of *S. aureus*-specific Treg expressed TIGIT, a pleiotropic immune checkpoint. Indeed, blocking TIGIT with Tiragolumab, an antagonistic anti-TIGIT mAb, increased IL-1β production in response to *S. aureus in vitro* stimulation.

Overall, these findings provide a high-resolution characterization of *S. aureus* pre-existing CD4^+^ T cell response with implications for future vaccine and therapeutic design.

## Materials and methods

### Peripheral blood mononuclear cells

PBMCs isolated from buffy coats from healthy donors by density gradient centrifugation, frozen in fetal bovine serum (FBS) supplemented with 10% DMSO and kept frozen at -150°C until use, were obtained from Tivoli Hospital (Brussels, Belgium).

### Preparation of heat-killed bacteria

*S. aureus* USA300, LAC strain, and *Klebsiella pneumoniae*, 9163 O2a strain, were grown in tryptic soy broth to exponential phase reaching an optical density (OD)_600_ of 0.6 and 1 respectively. Secreted bacterial proteins were removed by washing in sterile PBS, then bacteria were heat-inactivated for 45 min at 90°C. Next, bacterial particles were washed 3 times in PBS and protein concentration was determined by using the Pierce BCA protein assay (Thermo Fisher Scientific) according to manufacturer’s instructions. CFU counts were then estimated using the previously reported formula: 25 µg bacteria ≈ 1 x 10^8^ CFU (28). Heat-inactivation was confirmed by spreading bacterial suspension on Tryptic Soy Agar plates and incubation at 37°C overnight. No bacterial growth was detected.

### AIM assay

Frozen PBMCs were thawed in prewarmed Ca^2+^- and Mg^2+^-free PBS (Gibco) containing 2.5 mM EDTA (Sigma) and 20 μg/ml Dnase I (Sigma), washed and counted using the Vi-CELL XR cell counter (Beckman Coulter). PBMCs were then resuspended in separation buffer (PBS with 2 mM EDTA, 0.5% BSA from Sigma), and CD4^+^ T cells were negatively selected using the MACS CD4^+^ negative isolation kit (Miltenyi Biotec) following manufacturer’s instructions. The CD4^-^ fraction was recovered and irradiated with 30 Gy. CD4^+^ and CD4^-^ fractions were washed and resuspended at a concentration of 1 x 10^6^ cells/ml in c-RPMI (RPMI-1640 supplemented with 1% minimum essential medium non-essential amino acids, 1% penicillin-streptomycin-glutamine, 1% sodium pyruvate (all from Gibco), and 5% FBS (Hyclone). Both fractions were recombined at a ratio of 1:1 and 2 x 10^5^ total cells were plated in a 96-well round bottom plate (Corning) and stimulated with HK *S. aureus*, HK *K. pneumoniae* or HK *C. albicans* (ATCC10231, Invitrogen) at a multiplicity of infection of roughly 3:1 or recombinant SARS-CoV-2 spike protein and the chemically-inactivated tetravalent influenza vaccine Influvac S tetra (Abbott) at a final concentration of 2 µg/ml. For comparison, cells were stimulated polyclonally using plate-bound anti-CD3 antibody (clone OKT3, BD Bioscience, cat # 566685, 1 µg/ml) and soluble anti-CD28 antibody (clone CD28.2, BD Bioscience, cat # 555725, 2 µg/ml). As a negative control, cells were left unstimulated with c-RPMI (Medium). Cells were incubated for 24 h at 37°C, 5% CO_2_.

### SORT-Seq of AIM^+^ CD4^+^ T cells

Cells were washed twice and multiple wells from the same culture condition were pooled into polypropylene FACS tubes (Corning) for single cell sorting using BD FACS-Aria Fusion. CD3^+^CD4^+^CD8^-^ live cells were single-cell sorted based on the co-expression of CD137 and OX40 (AIM^+^ cells) into precast 384-well plates (Single Cell Discoveries, Utrecht, The Netherlands). Index sorting for CLA was applied to correlate the expression of this surface marker to the transcriptional profile at the single cell level. After sorting, plates were immediately spun and placed on dry ice, stored at -80°C and then shipped on dry ice to Single Cell Discoveries. For each of the 6 healthy subjects analyzed, two plates for each stimulation condition (HK *S. aureus* or α-CD3/CD28) were analyzed by scRNA-seq according to an adapted version of the SORT-seq protocol (36).

### Surface staining for flow cytometry and index-cell sorting

Cells were washed twice with PBS and stained with a viability dye (see **Supplementary Table S1**) for 20 min at RT. Samples were washed, blocked either with 2% rabbit serum in PBS or Fc Block on ice for 20 min. Surface staining for various surface expressed markers was carried out for 20 min on ice. For non-sorting related experiments cells were read using a BD FACSymphony A3.

### Analysis of SORT-seq data

SORT-seq data were analyzed using the scanpy package on Python. For Quality Control (QC), first doublets were removed using the scanpy implementation of Scrublet (37) and a threshold of 0.25. Then, cells that had fewer than 1,000 genes being expressed, and genes that appear in fewer than 4 cells were removed. Also, cells with more than 6,000 total counts were considered outliers after manual inspection and discarded. Finally, cells with a percentage of mitochondrial genes above 25% were discarded to avoid introducing bias. After QC, we retrieved 1,791 cells from the HK *S. aureus* stimulation and 3,032 cells from the α-CD3/CD28 stimulation for a total of 4,823 cells. Highly-variable genes were identified using the raw counts without normalization using the scanpy ‘highly_variable_genes’ script with the ‘seurat_v3’ flavor to account for the different datasets. To compare different cells, the counts were normalized to 10K, and then a logarithm transformation was applied to compute the PCA, the neighbors with 6 principal components, and the UMAP. To correct for different batches, we applied the bbknn implementation of the batch balanced neighbors algorithm (38). The scanpy implementation of the Leiden algorithm (39) was used to identify 4 clusters with a resolution of 0.2 after visual inspection. A small cluster of 105 cells was removed at this stage as it was not expressing genes related to a CD4^+^ T cell population, reducing the number of clusters to 3 as reported in the main text. GSEA analysis was conducted with the Python package ‘gseapy’ as described in (40). Trajectory analysis followed closely the protocol explained in the tutorial of the Python package scFates (41). Briefly, we identified a spanning tree of 200 nodes using the Python package ‘palantir’ to estimate the neighboring graph, and then labelled the node closest to center of the UMAP as the root. We used the scfates functions ‘test_association’ and ‘fit’ to compute the genes associated with the trajectories and their pseudotime, as reported in **Figure 2C**.

**Figure 1:**
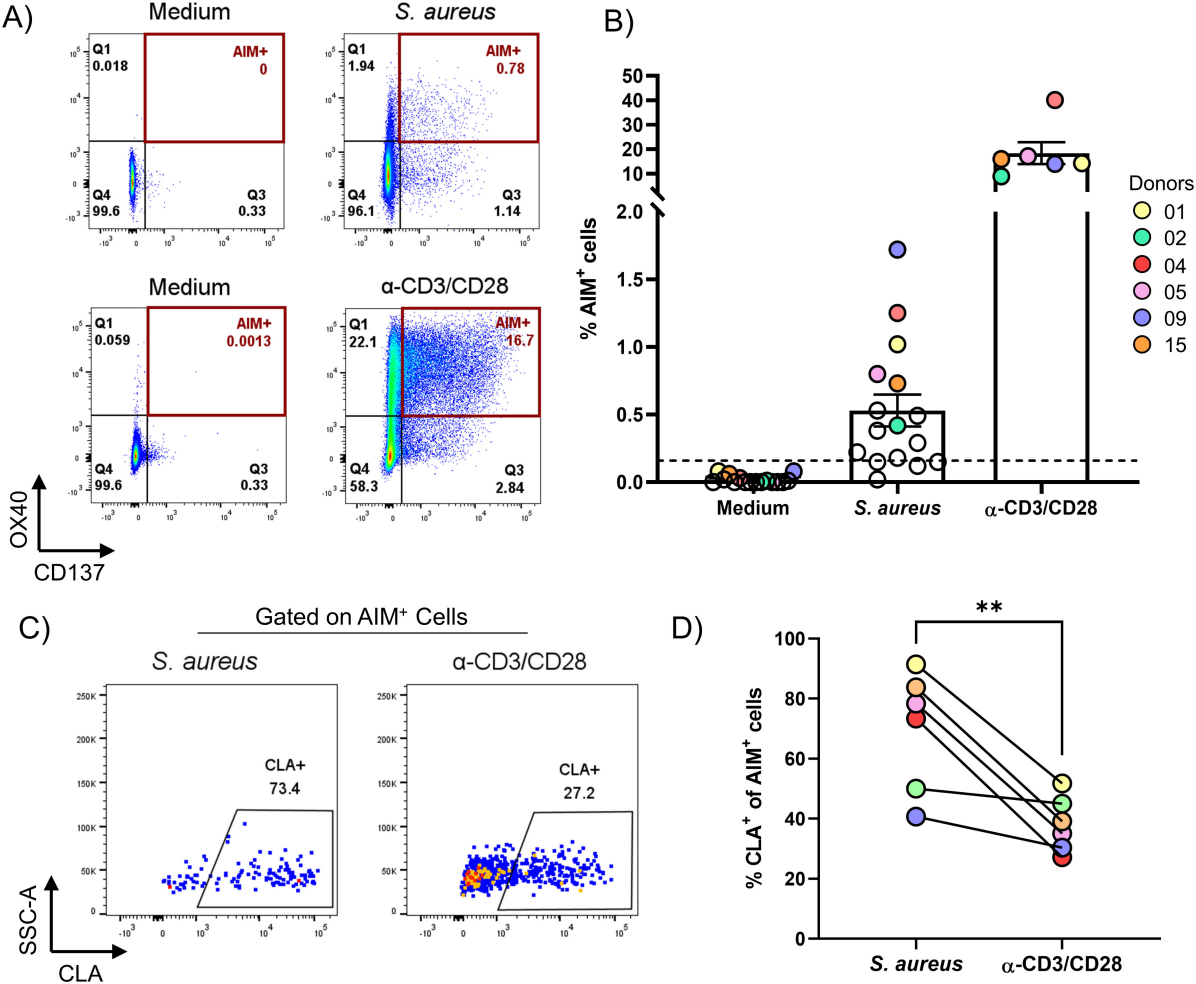
AIM assay identified CD4^+^ T cells specific for *S. aureus* that are enriched for the skin-homing marker CLA in the blood of healthy subjects. PBMCs from healthy donors were enriched via negative selection into CD4^+^ T cells (CD4^+^) and CD4^+^ T cell-depleted (CD4^-^) fractions. CD4^-^ fraction was irradiated and mixed at a 1:1 ratio with the CD4^+^ fraction. Cells were then stimulated for 24 h with HK *S. aureus*, or α-CD3/CD28 as positive control, or left untreated (Medium) as negative control. **(A)** Flow-cytometric plots showing co-expression of OX40 and CD137 (AIM^+^) on CD4^+^ T cells from one representative donor after stimulation with HK *S. aureus* (*S. aureus*-specific) or α-CD3/CD28 antibodies (polyclonally-activated). See **Supplementary Figure 1** for gating strategy. **(B)** Percentages of AIM^+^ CD4^+^ T cells for 16 healthy donors analyzed. Each circle represents a donor, colored circles represent the 6 donors that were further analyzed for CLA expression in D and by SORT-seq. Mean values ± 95% CI for each culture condition are also shown. Dotted line represents the arbitrary cut-off (0.16% AIM^+^ CD4^+^ T cells, set doubling the highest percentage of AIM^+^ CD4^+^ T cells observed without stimulation) used to identify responders to *S. aureus* (12 donors out of 16). **(C)** Flow-cytometric plots showing CLA expression on *S. aureus*-specific and polyclonally-activated CD4^+^ T cells from one representative donor. **(D)** Donor-matched percentages of *S. aureus-*specific or polyclonally-activated CLA^+^CD4^+^ T cells from 6 donors (indicated by colored circles in **B**). Statistical analysis was performed using a paired t-test. *P* ≤ **0.01.

**Figure 2:**
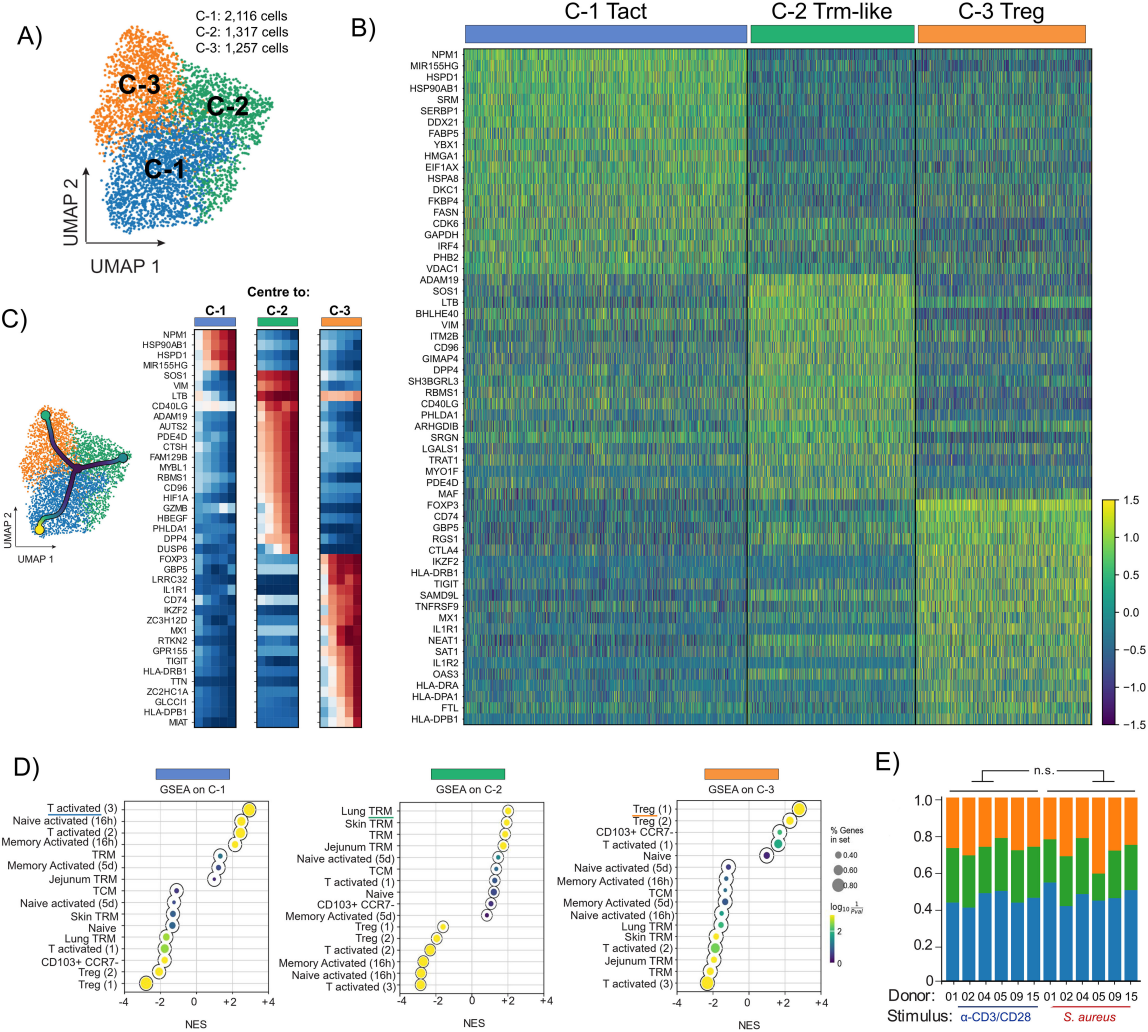
SORT-Seq analysis of *S. aureus*-specific and polyclonally-activated CD4^+^ T cells revealed 3 cell clusters: Activated T cells (Tact), Trm-like cells and Treg. PBMCs isolated from 6 healthy donors (coloured circles in **Figures 1B, D**) were separated via negative selection into CD4^+^ and CD4^-^ fractions. After irradiation of CD4^-^ cells, both fractions were re-mixed at a 1:1 ratio and stimulated with HK *S. aureus* or polyclonally with α-CD3/CD28. CD4^+^ T cells co-expressing OX40 and CD137 (AIM^+^) after 24 h stimulation were sorted via flow-cytometry and analyzed by SORT-seq. **(A)** UMAP representation of scRNA-seq merged data of *S. aureus*-specific and polyclonally-activated CD4^+^ T cells from 6 donors (4,690 cells in total). Merged datasets were divided into 3 clusters via Leiden clustering and annotated using different colors. **(B)** Heatmap of unsupervised clustering analysis showing the fold upregulation of the top 20 genes used to differentiate each of the 3 clusters shown in **(A, C)** Trajectory analysis starting from CCR7-expressing CD4^+^ T cells (black dot in the center of the UMAP). Heatmap showing that cells along the trajectory displayed signature differences increasingly associated with different cell function of Tact, Trm-like and Treg cells. **(D)** Gene set enrichment analysis (GSEA) reporting the Normalized Enrichment Score (NES) for each of the 3 clusters using the top 50 cluster-defining genes compared against gene lists obtained from published literature. Circle size indicates proportion of genes present in a specific gene set whereas the color scale refers to P value. **(E)** Proportional representations in each of the 3 clusters for each of the 6 donors analyzed per stimulation condition. No significative difference by two-tailed t-test among *S. aureus*-specific and polyclonally-activated CD4^+^ T cells.

### Sorting and intracellular cytokine staining of *S. aureus-*specific and polyclonally-activated CD4^+^ T cells

HK *S. aureus* stimulated cells AIM^+^ and AIM^-^ (OX40^-^ CD137^-^) and α-CD3/CD28 stimulated AIM^+^ cells were sorted into 5 ml FACS-tubes containing c-RPMI. Cells were then re-suspended in 200 µl, plated in a 96-well round bottom plate, rested for 14 h when cells were treated with PMA (50 ng/ml) and Ionomycin (1 µM). After 1 h, Brefeldin A was added and cells were incubated for an additional 3 h. After stimulation cells were washed twice with PBS, stained with Live/Dead UV440 (ThermoFisher Scientific), washed with PBS and blocked using FcBlock (BD). Then, cells were surface stained for CD4 and CLA for 20 min on ice. After washing twice in PBS, cells were fixed and permeabilized using Cytofix/Cytoperm (BD) for 20 min on ice. Cells were then washed twice with Perm/wash buffer (BD) and stained intracellularly for IFN-γ and IL-17A for 15 min at RT. Cells were washed twice and then read on a BD FACSymphony A5.

### Anti-TIGIT treatment

Isolated CD4^+^ T cells were recombined with CD4^-^ at a ratio of 1:1 and 2 x 10^5^ total cells were plated in a 96-well round bottom plate (Corning). The antagonistic anti-TIGIT antibody Tiragolumab or an isotype-matched control (both from MedChemExpress), each at a final concentration of 20 µg/ml, or just medium were added to the cultures 15 min before stimulation with HK *S. aureus* (MOI 3) or no stimulation. After 3 d, concentrations of secreted cytokines in the culture supernatants were measured by V-PLEX Human Cytokine 36-Plex Kit (Meso Scale Discovery, as previously described (42).

### Statistics

**Figure 3:**
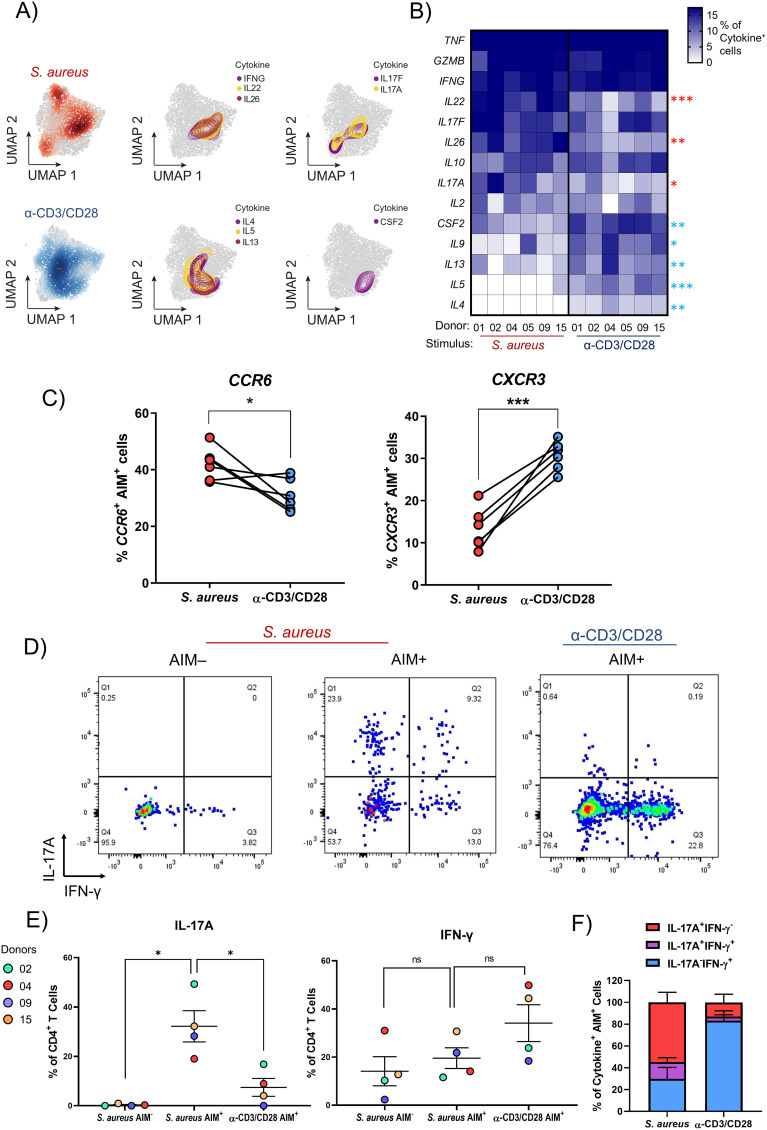
The cytokine signature of *S. aureus*-specific CD4^+^ T cells from healthy donors consists of IL17A, IL22, and IL26. **(A-C)** PBMCs isolated from 6 healthy donors were sorted via negative selection into CD4^+^ and CD4^-^ fractions. CD4^-^ cells were irradiated and both fractions were re-mixed at a ratio of 1:1 and stimulated for 24 h with HK *S. aureus* or α-CD3/CD28. AIM^+^ CD4^+^ T cells were then FACS-sorted and scRNA-seq analysis performed. **(A)** UMAPs showing the distinct cell localizations for each stimulation condition: *S. aureus*-specific and polyclonally-activated CD4^+^ T cells in red and blue, respectively. The distribution and co-localization of cytokine transcription was assessed by mapping areas of expression (represented by colored contours) for the reported cytokine-encoding genes. **(B)** Heatmap representing the percentages of *S. aureus*-specific and polyclonally-activated CD4^+^ T cells transcribing a panel of 14 cytokines calculated for each donor. Cytokines were listed from the one expressed by the highest to the one expressed by the lowest percentage of *S. aureus*-specific CD4^+^ T cells. Cytokine genes expressed by statistically higher percentages of *S. aureus*-specific vs. polyclonally-activated CD4^+^ T cells are indicated by red asterisks while those expressed by higher percentages of polyclonally-activated cells are indicated by blue asterisks. **(C)** Each symbol represents the percentage of *S. aureus*-specific or polyclonally-activated CD4^+^ T cells transcribing *CCR6* or *CXCR3* from 1 out of 6 donors analyzed. Mean values ± SEM are also shown. **(D-F)** Three distinct cell populations were FACS-sorted from 4 donors: after HK-*S. aureus* stimulation OX40^-^CD137^-^ (AIM^-^) and AIM^+^ CD4^+^ T cells, and after α-CD3/CD28 stimulation AIM^+^ CD4^+^ T cells. Cells were rested overnight, stimulated with PMA/Ionomycin for 4 h and stained intracellularly for IL-17A and IFN-γ. **(D)** Dot plots from a representative donor gating on live, CD4^+^ T cells. **(E)** Percentages and **(F)** Boolean gates of IFN-γ- and/or IL-17A-producing CD4^+^ T cells from 4 donors. Statistical analysis was performed using paired students t-tests **(B, C)** or a one-way ANOVA with Dunnett’s test for multiple comparisons **(E)**. *P ≤ 0.05, **P ≤ 0.01, *P* ≤ ***0.001.

**Figure 4:**
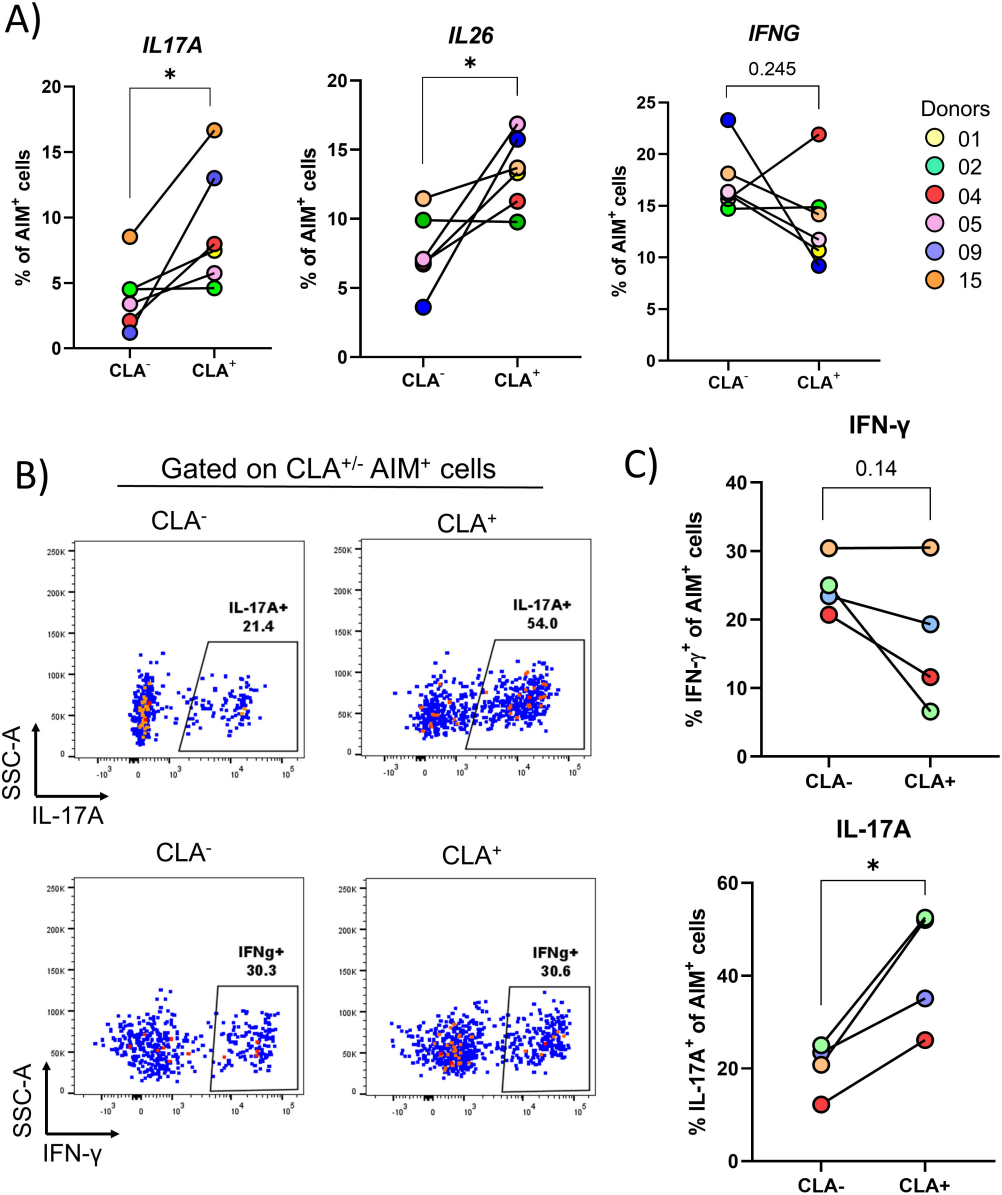
Skin-tropic *S. aureus*-specific CD4^+^ T cells are further enriched for IL17A and IL26 gene expression. PBMCs isolated from 6 healthy donors were sorted via negative selection into CD4^+^ and CD4^-^ fractions. CD4^-^ cells were irradiated and both fractions were re-mixed at a ratio of 1:1. Cells were then stimulated for 24 h with HK *S. aureus* or α-CD3/CD28 or left untreated. CD4^+^ T cells expressing the activation markers OX40 and CD137 (AIM^+^ CD4^+^ T cells) were index-sorted based on CLA expression via flow-cytometry and scRNA-Seq analysis was performed. **(A)** The transcription of the 14 cytokine-encoding genes analyzed in **Figure 3B** was quantified in *S. aureus-*specific CD4^+^ T cells separated based on CLA expression. Only *IL17A* and *IL26* were transcribed at statistically significantly higher levels in CLA^+^ as compared to CLA^-^ cells, as assessed by paired Student’s t-test. *P ≤ 0.05. To confirm transcriptional results at the protein level, AIM^+^ CD4^+^ T cells after stimulation with HK *S. aureus* were FACS-sorted, rested overnight and then re-stimulated with PMA and Ionomycin for 3 h and stained on surface for CLA and intracellularly for IL-17A and IFN-γ. **(B)** Dot plots from a representative donor are shown. **(C)** Percentages of IL-17A^+^ or IFN-g^+^ cells in CLA^+^ and CLA^-^ cells of 4 donors are shown. Statistical analysis was performed using paired Student’s t-test. *P ≤ 0.05.

**Figure 5:**
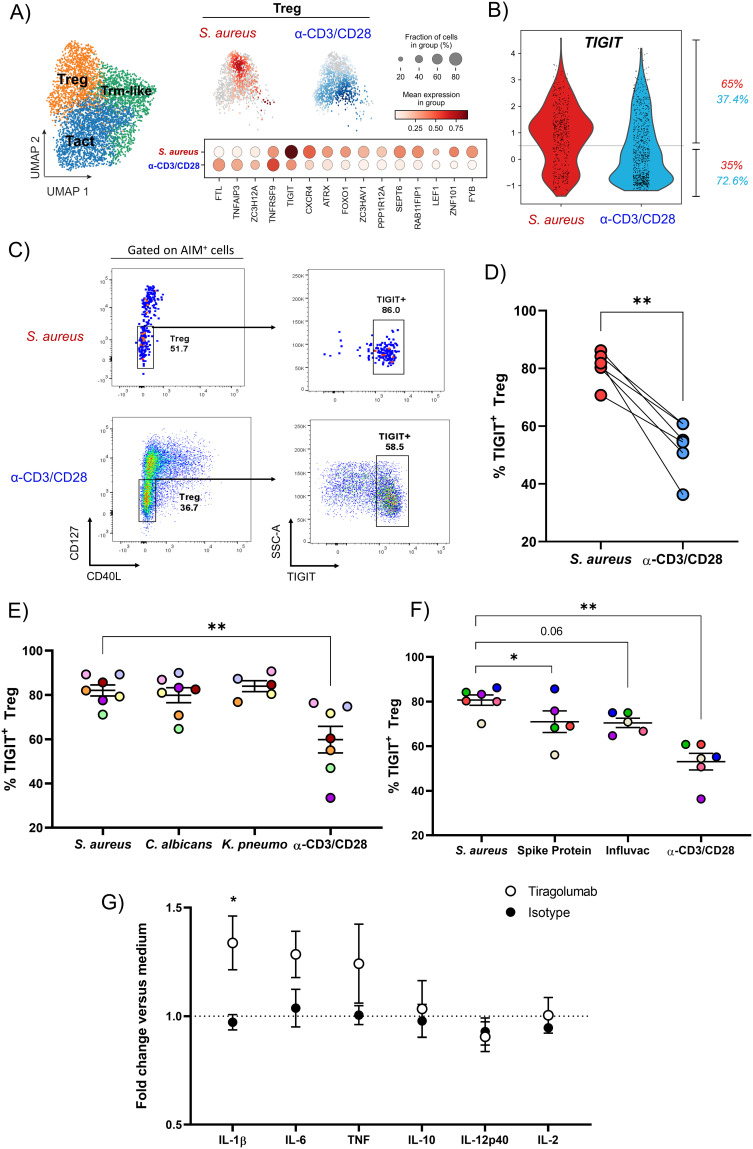
*S. aureus*-specific Treg present in the blood of healthy subjects express the co-inhibitory receptor TIGIT. **(A)** Selecting cells from the Treg cluster (in orange in the UMAP), the differentially expressed genes between *S. aureus*-specific (in red) and polyclonally-activated (in blue) Treg are reported. **(B)** Violin plot showing on a cell per cell basis the expression of *TIGIT* in *S. aureus*-specific and polyclonally-activated Treg. Percentages of cells expressing *TIGIT* in each group are reported. **(C, D)** Surface expression of TIGIT on *S. aureus*-specific and polyclonally-activated Treg identified as CD40L^-^CD127^low^ CD4^+^ T cells. **(C)** Representative dot plots gating on AIM^+^ cells after *S. aureus* or α-CD3/CD28 stimulation. **(D)** Percentages of AIM^+^ Treg expressing TIGIT in response to each stimulus for 6 donors. **(E, F)** Percentages of AIM^+^TIGIT^+^ Treg after stimulation with **(E)** HK microorganisms: *S. aureus* (*n* = 6 donors)*, Candida albicans* (*C. albicans*, *n* = 6 donors), *Klebsiella pneumoniae* (*K. pneumoniae*, *n* = 4 donors), or **(F)** SARS-Cov2 Spike Protein, Influvac Tetra vaccine (*n* = 5 donors/stimulus). Polyclonal stimulation with α-CD3/CD28 was done for comparison (*n* = 6 donors). **(G)** CD4^+^ fraction mixed with irradiated CD4^-^ fraction from PBMCs of healthy subjects were stimulated with HK *S. aureus* as described for AIM assay for 3 d in presence of the antagonist anti-TIGIT mAb Tiragolumab, isotype control mAb or medium alone. Concentrations of cytokines present in cultures supernatants collected at day 3 were measured and expressed as fold induction versus concentrations found in cultures where no antibodies were added (medium, dotted line, *n* = 4-5 donors, 3 independent experiments). Statistical analysis was performed using a paired t-test **(D)**, or a mixed-effects analysis followed by Dunnet’s multiple comparison test **(E, F)** or a 2-way ANOVA followed by Sidak’s test for multiple comparisons **(G)**. *P ≤ 0.05, *P* ≤ **0.01.

Comparisons between different cell populations at the transcriptional level were tested using the paired t-test as the hypothesis of a normal distribution could not be discarded according to the Shapiro-wilk test (**Figures 2E**, **3B, F**, **4A**). Gene signatures were derived using the scanpy function ‘rank_genes_groups’ with a Wilcoxon rank-sum method as suggested in (43) (**Figures 2B**, **5A**). GraphPad Prism 8.0.1 was used to perform other statistical analyses specified in figure legends.

## Results

### AIM assay identified CD4^+^ T cells specific for *S. aureus* that are enriched for the skin-homing marker cutaneous lymphocyte-associated antigen in the blood of healthy subjects

AIM assays can track T cell responses specific for complex stimuli, like whole microbes or lysates. In addition, AIM assays can identify and phenotype a wider breadth of antigen‐specific T‐cell responses than proliferation or cytokine‐based assays. Numerous cell surface receptors have been authenticated as being able to identify human antigen–specific CD4^+^ and CD8^+^ T cells (44). Among them, to identify pre-existing *S. aureus* specific CD4^+^ T cells in the blood of healthy individuals, an AIM assay based on the co-expression of OX40 (CD134) and CD137 (4-1BB) was used (45). Importantly, co-expression of these markers is dependent on T cell-receptor contact with the cognate antigen expressed on antigen presenting cells (APCs) and independent of bystander activation [data not shown (46)]. This AIM assay was performed to screen 16 healthy subjects for the presence of *S. aureus*-specific CD4^+^ T cells in the blood. To do so, CD4^+^ T cells were negatively enriched from PBMCs and mixed 1:1 with the irradiated positively selected non-CD4^+^ T (CD4^-^) cells. To identify *S. aureus*-specific CD4^+^ T cells, cultures were stimulated with heat-killed (HK) *S. aureus*, which contains denatured proteins present in live bacteria but not secreted proteins including toxins, for 24 h. As a positive control, cultures were stimulated polyclonally with a combination of anti-CD3 and anti-CD28 antibodies (α-CD3/CD28). As shown in **Figures 1A, B**, a sizable portion of CD4^+^ T cells co-expressed OX40 and CD137 (AIM^+^ cells) in response to *S. aureus* (0.529 ± 0.470, mean ± SD) in the vast majority of the subjects analyzed (75%: 12 responders out of 16 donors tested, **Figures 1A, B**). These cells are referred to as *S. aureus*-specific CD4^+^ T cells. The observed variability in frequencies of *S. aureus*-specific CD4^+^ T cells among donors likely reflects the individual history of interactions with *S. aureus*.

Our previous work has shown that *S. aureus*-specific CD4^+^ tissue-resident memory T cells (Trm), which express the skin-homing marker CLA, are abundant in the skin of healthy subjects (11), suggesting that the skin is a primary site of encounter with *S. aureus*. Elegant studies have shown that human Trm can leave the skin (ex-Trm) and re-enter the circulation (47–49), keeping the expression of CLA, which confers them skin-tropism. Therefore, circulating *S. aureus*-specific AIM^+^ cells were analyzed for the expression of CLA. As shown in **Figures 1C, D**, much higher percentages of *S. aureus*-specific CD4^+^ T cells expressed CLA as compared to cells polyclonally-activated with α-CD3/CD28 (69.6 ± 19.9 vs. 38.1 ± 9.2, mean ± SD). This finding reinforces the notion that the pre-existing memory CD4^+^ T cell response to *S. aureus* is biased towards a skin-homing phenotype.

### Single-cell transcriptomic analysis revealed 3 distinct populations of pre-existing CD4^+^ T cells specific for *S. aureus* in the blood of healthy subjects: Activated T cells, Trm-like cells and Treg

To gain a high-resolution and effector function-independent understanding of the pre-existing CD4^+^ T cell response to *S. aureus* in the blood of healthy subjects we performed single-cell RNA sequencing (scRNA-seq) via SORT-seq on AIM^+^ cells specific for *S. aureus* from 6 pre-selected donors (shown in color in **Figure 1B**). To establish the signature of *S. aureus*-specific CD4^+^ T cells, SORT-seq was also performed on AIM^+^ cells from the same donors activated polyclonally by α-CD3/CD28 antibodies. Since CLA derives from glycosylation of P-selectin glycoprotein ligand-1 (PSGL-1), to track skin-tropic AIM^+^ cells index sorting for CLA^+^ was performed. Through Uniform Manifold Approximation and Projection (UMAP) analysis and Leiden clustering, sequenced cells that passed QC filtering (4,690 cells in total) were projected in two dimensions and designated into 3 clusters based on the individual transcriptome of each cell (**Figure 2A**). Remarkably, the clusters were contiguous reflecting the fact that the functional states of CD4^+^ T cells is a continuum rather than defined Th subtypes. The top 20 genes more differentially expressed in each cluster are shown (**Figure 2B**). Despite the high similarity in gene expression between the three clusters, trajectory analysis revealed a gradient of gene activation (**Figure 2C**). Gene set enrichment analysis (GSEA) was then performed using gene sets from previously published work in which human CD4^+^ T cells from various tissues and in various activation states were transcriptionally defined (50, 51). Significantly positive net enrichment scores (NES) were obtained for activated T cells for cluster 1 that was therefore annotated as Tact, while cluster 2 showed significant matches for subsets of Trm. Given that the analyzed cells were derived from peripheral blood, cluster 2 was annotated as Trm-like. Finally, significantly positive NES values were obtained between cluster 3 and Treg populations, which was annotated as Treg (**Figure 2D**). Of note, although the proportions of *S. aureus*-specific and polyclonally-activated CD4^+^ T cells in the 3 clusters identified were comparable with minor variability among donors (Tact: 46.9% ± 4.5 *vs*. 44.8% ± 3.4; Trm-like: 24.3% ± 5.3 *vs*. 28.0% ± 1.5; Treg: 28.0% ± 7.2 *vs*. 27.1% ± 3.1, mean ± SD for *S. aureus*-specific *vs*. polyclonally-activated CD4^+^ T cells, **Figure 2E**), *S. aureus*-specific and polyclonally-activated CD4^+^ T cells occupied different areas of the UMAP (**Figure 3A**, shown in red and blue, respectively).

### *S. aureus*-specific CD4^+^ T cells are enriched for cells that express the Th17-type cytokines encoding genes *IL-17A*, *IL-22* and *IL-26* as compared to polyclonally-activated CD4^+^ T cells

To better capture differences in the phenotypes of *S. aureus-*specific or polyclonally-activated CD4^+^ T cells, the transcriptional expression of 14 cytokines was analyzed. When spatially embedded onto UMAP projections, patterns of co-localization between certain cytokines, indicating cytokine co-expression by either the same cells or cells with a similar phenotype, were observed (**Figure 3A**). This was the case for *IL17A* and *IL17F*, which showed co-localization in two regions of the UMAP, located mainly in the Tact cluster, in areas where *S. aureus*-specific (in red) were more abundant than polyclonally-activated (in blue) CD4^+^ T cells. *IFNG, IL22* and *IL26* co-localized in the Tact cluster, in a region enriched for *S. aureus*-specific cells where *IL17A* and *IL17F* co-localized, too. On the other hand, cells expressing the Th2-type cytokine-encoding genes *IL4, IL13* and *IL5* co-localized mainly in a different region of the Tact cluster, enriched for polyclonally-activated cells, where cells expressing *CSF2* (encoding GM-CSF) were also concentrated. To quantify differences in the expression of these 14 cytokine-encoding genes between *S. aureus*-specific and polyclonally-activated CD4^+^ T cells, a heatmap showing the percentage of cells expressing each cytokine in response to each stimulus was generated across each donor (**Figure 3B**). Although donor-to-donor differences were observed, overall, the expression of genes encoding IL-22, IL-26 and IL-17A, all cytokines indicative of a Th17 phenotype (38, 39), was found in significantly higher percentages of CD4^+^ T cells specific for *S. aureus* as compared to polyclonally-activated cells. The same was true for the gene encoding CCR6, a chemokine receptor preferentially expressed by human Th17-type cells (52) (**Figure 3C**). Though expressed by even higher percentages of *S. aureus*-specific CD4^+^ T cells, genes encoding TNF, Granzyme B and IFN-g showed no enrichment when compared to polyclonally-activated CD4^+^ T cells. In contrast, percentages of cells expressing genes encoding the Th2-type cytokines IL-13, IL-5 and IL-4 as well as GM-CSF (encoded by *CSF2*) and IL-9, were significantly higher in polyclonally-activated as compared to *S. aureus-*specific CD4^+^ T cells, which expressed these genes at very low levels.

To validate these results at the protein level, *S. aureus-*specific and polyclonally-activated CD4^+^ T cells were FACS-sorted and analyzed by intracellular cytokine staining for IFN-γ and IL-17A after stimulation with PMA and Ionomycin. Consistent with results obtained at the transcriptional level, the percentages of AIM^+^ cells producing IL-17A were significantly higher in response to HK *S. aureus* than to polyclonal stimulation (32.2 ± 12.7 *vs*. 7.4 ± 7.2, mean ± SD) while the opposite trend was observed for IFN-γ (19.5 ± 8.6 *vs.* 34.1 ± 15.3) (**Figures 3D–F**). Interestingly, a considerable percentage of *S. aureus*-specific CD4^+^ T cells co-produced IL-17A and IFN-γ (**Figures 3D, E**).

Taken together these results demonstrated that a sizable portion of CD4^+^ T cells present in the blood of healthy subjects is poised to secrete IL-17A and/or IFN-g upon *S. aureus* encounter.

### Skin-tropic *S. aureus*-specific CD4^+^ T cells are enriched for the production of Th17-type cytokines

Having observed an enrichment for a skin-tropic phenotype (CLA expression, **Figure 1D**), the transcriptional profile of *S. aureus*-specific CD4^+^ T cells was analyzed based on surface CLA expression, as assessed by index sorting. Remarkably, the percentages of *S. aureus*-specific cells expressing the *IL17A* (2.3 fold increase, *P* = 0.031) and *IL26* (1.8 fold increase, *P* = 0.027), but not the *IFNG* (0.7 fold increase, *P* = 0.245), genes were significantly higher in CLA^+^ as compared to CLA^-^ cells (**Figure 4A**). These findings were validated performing intracellular staining for IL-17A and IFN-γ on FACS-sorted *S. aureus*-specific CD4^+^ T cells either CLA^+^ or CLA^-^. As shown in **Figures 4B, C**, a significant increase in the percentages of IL-17A-producing CD4^+^ T cells was observed in CLA^+^ *vs*. CLA^-^ cells (2.0 mean fold increase, *P* = 0.02). No such increase was found for IFN-γ, which showed a trend towards decreased production in skin-homing cells (0.7 mean fold increase, *P* = 0.14). Together these results demonstrate that skin-tropic *S. aureus*-specific CD4^+^ T cells present in the blood of healthy subjects display elevated production of Th17-type cytokines in line with the reported phenotype of *S. aureus*-specific Trm present in the skin of healthy subjects (11).

### Treg specific for *S. aureus* express the immunomodulatory protein TIGIT

To investigate the phenotype of *S. aureus*-specific Treg, differential gene expression analysis was performed on sequenced AIM^+^CD4^+^ T cells from Treg cluster *S. aureus-*specific or polyclonally-activated. *TIGIT*, which encodes for the negative immune checkpoint TIGIT, was among the most upregulated genes within *S. aureus*-specific Treg, 65% of which were actively transcribing *TIGIT* versus 37% of those polyclonally-activated (**Figure 5B**). This difference was confirmed by FACS analysis of TIGIT expression on *S. aureus*-specific or polyclonally-activated Treg, identified as CD127(IL-7R)^low^CD40L^-^ cells (**Figures 5C, D**) or as FoxP3^+^ cells (**Supplementary Figure 2**) (53).

To establish if TIGIT expression is a hallmark of *S. aureus*-specific Treg, TIGIT expression in circulating Treg specific for *Candida albicans*, *Klebsiella pneumoniae*, SARS-CoV-2 spike protein, or the seasonal influenza vaccine was assessed by gating on AIM^+^ Treg (CD4^+^CD127^low^CD40L^-^OX40^+^CD137^+^) after *in vitro* stimulation. While percentages of TIGIT^+^ Treg specific for *C. albicans* and *K. pneumoniae* were comparable to those specific for *S. aureus* (**Figure 5E**), those specific for SARS-CoV-2 spike protein or for an inactivated tetravalent influenza vaccine were significantly lower (**Figure 5F**). Taken together these findings suggest that TIGIT^+^ Treg may be particularly associated with memory CD4^+^ T cell responses to pathobionts.

In Treg, TIGIT induces the suppressive mediator fibrinogen-like protein 2 (Fgl2), which not only confers TIGIT^+^ Treg cells with superior suppressive function but also enables selectivity toward suppressing Th1 and Th17 (54). This allows TIGIT to shift the cytokine balance away from a proinflammatory Th1- and Th17-dominated response to restore homeostasis. Of note, *S. aureus*-specific, but not polyclonally-activated, Treg expressed *FGL2* encoding Fgl-2 (data not shown). In addition, TIGIT can deliver an inhibitory signal into the APCs that express CD155 (Poliovirus receptor, PVR), driving them to a more tolerogenic phenotype (55). Expression of TIGIT by the majority of *S. aureus*-specific Treg suggested that TIGIT could play a key role in the suppression exerted by these cells. To test this hypothesis, TIGIT function was blocked *in vitro* using Tiragolumab, an antagonistic anti-TIGIT monoclonal antibody currently under evaluation in a large number of clinical trials in cancer patients in combination with the anti-PD-L1 mAb Atezolizumab (55). Purified CD4^+^ T cells were cultured with irradiated CD4-depleted PBMCs and stimulated with HK *S. aureus* in the presence or absence of Tiragolumab or isotype-matched control antibody for 3 days. Culture supernatants were then harvested and analyzed for the production of a panel of cytokines. Addition of Tiragolumab induced a statistically significant increase in IL-1β production as compared to isotype control (1.34 ± 0.25 *vs.* 0.97 ± 0.07, mean fold induction *vs*. medium ± SD). Moreover, Tiragolumab treatment resulted in a non-significant increase in the production of TNF and IL-6 but had no effect on other cytokines such as IL-10, IL-2 and IL-12 (**Figure 5G**). These results suggest that pre-existing *S. aureus*-specific Treg may function through the expression of TIGIT to inhibit a pro-inflammatory response.

## Discussion

The successful translation of *S. aureus* vaccine efficacy from preclinical mouse models into human subjects has proved incredibly challenging. One major difference between mice and humans is that because of colonization interactions, humans commonly bear pre-existing immune memory for *S. aureus*, as a consequence of this organism propensity to asymptomatically colonize healthy humans, which does not occur in laboratory mice. The presence of memory CD4^+^ T cells that recognize *S. aureus* has been reported in the blood, skin and lungs of healthy subjects and classified as Th17 and/or Th1. It is likely that this pre-existing immunity or immune imprinting influences the outcome of the CD4^+^ T cell response induced by *S. aureus* vaccination and infection. Consequently, this study aimed to characterize the pre-existing *S. aureus*-specific CD4^+^ T cells present in the blood of healthy adults in a deep and unbiased way to capture the complexity of the memory CD4^+^ T cell responses occurring in humans (11, 13, 33). To achieve this goal, *S. aureus*-specific CD4^+^ T cells were single cell-sorted using an AIM assay based on co-expression of OX40 and CD137 induced by HK *S. aureus* stimulation and their transcriptome was analyzed by SORT-seq. Most importantly, this approach allowed to identify not only Tcon but also Treg. Therefore, this approach could be applied to other microbes and will be particularly useful to unveil the immune imprinting to other pathobionts, as shown here for *C. albicans* and *K. pneumoniae*. A detailed transcriptional analysis of cytokine production revealed further that the *S. aureus*-specific Tcon were enriched for a Th17-type response. Furthermore, for the first time, transcription of IL-26 and Granzyme B encoding genes was reported in *S. aureus*-specific CD4^+^ T cells adding greater resolution to the observed Th17-type phenotype. IL-17A and to a lesser degree, IL-22 have been linked to protection in mouse models of skin infection (27, 56) yet IL-26 remains quite understudied, likely due to its absence in the mouse genome and to the difficulty to reveal it by intracellular cytokine staining (49). Nonetheless, IL-26 is produced by human Th17 cells acting on epithelial cells and other immune cells to trigger inflammation (57–59) and even has direct antimicrobial activity against *S. aureus* (60). Indeed, the antimicrobial activity of IL-26 against *S. aureus* plays an important role in wound infections (60), while production of Granzyme B by Th17 cells specific for *Cutibacterium acnes* has been reported to be a means by which healthy skin commensals promote Th17-mediated host defense (61).

In studying *S. aureus*-specific CD4^+^ T cells, a large proportion of CLA^+^ cells that were further enriched for Th17-type cytokine production was observed. This finding is consistent with previous studies showing that *S. aureus*-specific CLA^+^CD4^+^ Trm cells are abundant in the skin of healthy subjects and are poised to produce IL-17A and IL-22 in response to *S. aureus* (11). Remarkably, in the current study *S. aureus*-specific CLA^+^ cells were selectively enriched in Treg (data not shown) suggesting that commensal interactions occurring at the skin induce a balanced Th17-type/Treg response sufficient to keep a low and superficial *S. aureus* load at bay under homeostatic conditions.

Notably, these findings suggest the ability to sample tissue-related T cell responses in blood of subjects and/or patients rather than in the tissue itself, in line with the capability of human skin-resident Trm cells to recirculate between skin and blood (49).

Given that *S. aureus* is capable of infecting multiple different bodily sites, and that in mice, diverse immune responses mediate protection towards *S. aureus* bacteremia, skin infection and pneumoniae, further understanding of tissue-specific immune signatures will be important for future vaccine development targeting different diseases (21, 62, 63).

To the best of our knowledge, this study characterizes Treg specific for *S. aureus* in human healthy subjects for the first time. Evidence for the presence of circulating *S. aureus-*specific Treg detected via an AIM assay based on CD137 expression on Treg has been reported, which we confirm, however no phenotypical analysis was performed previously (64). Treg are essential to establish and maintain immune homeostasis in peripheral tissues. Treg are primed by microbiota in neonates to promote tolerance towards them later in life. The induction of Treg by neonatal encounter with *S. aureus* has been hypothesized based on the fact that infants who harbored *S. aureus* in the gut during the first weeks of life had a decreased risk of developing food allergy as compared to children devoid of this bacterium (65). In addition, *in vitro* stimulation of neonatal CD4^+^ Tcon with *S. aureus* resulted in *de novo* generation of FoxP3^+^CD25^+^CD127^low^ cells (66). Elegant mouse studies have shown that the host is capable of differentiating between antigens expressed by *S. epidermidis* versus *S. aureus* during early-life skin colonization and mounts a preferentially tolerogenic response to the former. Hla toxin-mediated activation of IL-1R signaling upon *S. aureus* encounter was shown to be key in limiting enrichment of *S. aureus*-specific Treg (67). However, while Hla expression was presumably sustained in mice neonatally colonized using repeated topical associations with high bacterial loads (10^8^-10^9^ CFUs), since Hla is a toxin regulated by quorum sensing mechanisms, at low bacterial densities, as likely found during human neonatal colonization, Hla expression would be minimal or absent favoring Treg development.

Through comparative phenotypical analysis with polyclonally-activated Treg, TIGIT emerged as an overly expressed immunosuppressive marker on *S. aureus*-specific Treg. TIGIT has multiple immunoregulatory functions: i) ligation of CD155 (PVR) expressed on APCs is known to suppress proinflammatory cytokine secretion while inducing an immunoregulatory dendritic cell phenotype (68), ii) out competing the costimulatory molecule CD226 expressed on T cells thereby preventing T cell activation (69), iii) directly inhibiting intrinsic T cell activation (70). Indeed, immune regulation through TIGIT^+^ Treg plays a central role in anti-tumor immunity as loss of TIGIT on Treg, but not on other T cells, restores Tcon functions and slows tumor growth in preclinical cancer models (55).

In response to HK *S. aureus* stimulation, blocking TIGIT with the antagonist monoclonal antibody Tiragolumab, an increase in IL-1β secretion and a trend toward an increase in IL-6 and TNF secretion was observed. Notably, no such effect was present under polyclonal stimulation affirming that such suppression was *S. aureus*-specific (data not shown). As such, it is posited that *S. aureus*-specific Treg function via TIGIT to bind CD155 expressed on APCs thereby suppressing proinflammatory cytokine secretion. Further elucidation of TIGIT mechanism of action will be the object of future studies. Perhaps surprisingly, no changes were observed in the production of IL-10, IL-2 and IL-12 upon TIGIT blockade. While previous reports have shown that TIGIT can drive reduced IL-12 secretion with a concomitant increase in IL-10, this was shown to be stimulus dependent (68). Another reason behind the lack of effect of TIGIT treatment on the mentioned cytokines could be the selection of a non-optimal timepoint (72 hours) at which cytokines were analyzed.

Increasing IL-1β production towards *S. aureus* antigens could have important implications for vaccine development and *S. aureus* infection management. Firstly, IL-1β is an essential cytokine for the induction of *de novo* Th17 responses that, as previously described, are protective to *S. aureus* mucocutaneous infections in both mice and humans (71, 72). Secondly, IL-1β has been shown to inhibit IL-10 production by memory Th17 cells (73). Therefore, IL-1β may act not only at priming in lymphoid organs but also at the effector phase in target tissues. In addition, TIGIT^+^ Treg have been shown to selectively suppress the induction of Th17 and Th1 responses (54). It can therefore be hypothesized that *S. aureus*-specific Treg-mediated suppression during *S. aureus* vaccination will and has already hindered vaccine efficacy during clinical trials. Combining immune checkpoint inhibition (ICI) therapy with vaccination is currently the subject of intense and exciting research, particularly in the field of cancer vaccination (74). Indeed, cancer vaccine efficacy can be boosted using anti-PD-1 therapy in mice (75). Furthermore, subjects undergoing PD-1 therapy were found to have higher levels of seroconversion during influenza vaccination than those receiving vaccination alone (76). In this context anti-TIGIT therapy may function as a type of adjuvant acting to boost *S. aureus*-induced vaccine responses. Secondly, the cytokine IL-1β has been evidenced as essential for protection against *S. aureus* infections in multiple mouse models (77–80). Furthermore, in a clinical setting, lower serum levels of IL-1β were shown to be detrimental in the course of *S. aureus* bacteremia (81). As such, Treg may dampen effector immune responses in patients with *S. aureus* infection that could be ameliorated through the blocking of TIGIT. Notably, a recent study demonstrated that prevalence of TIGIT^+^ Treg in patients with COVID-19 correlated with negative clinical outcomes and with the development of bacteremia (82). Any future research investigating the applications of boosting IL-1β through anti-TIGIT therapy must however first understand any associated risks from overactive immune-responses as treatment with ICI can be accompanied by immune-related adverse events, mostly affecting sites colonized by the microbiota such as the skin and gastrointestinal tract. Recently, it has been shown in a mouse model that ICI treatment can unleash aberrant commensal-specific T cell responses leading to local inflammation and pathology (83).

It should be noted that some differences in observed cell phenotype between *S. aureus* and polyclonally activation may relate to different cell types being activated in each condition. Specifically, CD4^+^ T cells recognizing microbes such as *S. aureus* will primarily be of a memory phenotype whereas CD3/CD28 stimulation will act upon both naïve and memory T cell subsets.

To the best of our knowledge this study presents the first characterization of both conventional and regulatory CD4^+^ T cells specific for *S. aureus* in the blood of healthy subjects. A bias towards Th17-type and skin-tropic cells was shown and further, a population of Treg expressing the immunoregulatory receptor TIGIT was characterized. Lastly, this study identifies TIGIT as a biomarker that could be targeted to relieve the immunosuppressive response during vaccination proving beneficial for next-generation vaccines against *S. aureus* that aim to generate protective CD4^+^ T cell responses.

## Data Availability

The scRNA-seq data is publicly available in the Gene Expression Omnibus (GEO). The accession number is GSE285086.

## References

[1] Ikuta KS Swetschinski LR Robles Aguilar G Sharara F Mestrovic T Gray AP. Global mortality associated with 33 bacterial pathogens in 2019: a systematic analysis for the Global Burden of Disease Study 2019. Lancet. (2022) 400:2221–48. doi: 10.1016/S0140-6736(22)02185-7 PMC9763654 36423648

[2] Murray CJL Ikuta KS Sharara F Swetschinski L Robles Aguilar G Gray A. Global burden of bacterial antimicrobial resistance in 2019: a systematic analysis. Lancet. (2022) 399:629–55. doi: 10.1016/S0140-6736(21)02724-0 PMC8841637 35065702

[3] Clegg J Soldaini E McLoughlin RM Rittenhouse S Bagnoli F Phogat S. Staphylococcus aureus vaccine research and development: the past, present and future, including novel therapeutic strategies. Front Immunol. (2021) 12. doi: 10.3389/fimmu.2021.705360 PMC8294057 34305945

[4] Congdon ST Guaglione JA Ricketts OMA Murphy KV Anderson MG Trowbridge DA. Prevalence and antibiotic resistance of Staphylococcus aureus associated with a college-aged cohort: life-style factors that contribute to nasal carriage. Front Cell Infect Microbiol. (2023) 13. doi: 10.3389/fcimb.2023.1195758 PMC10333693 37441241

[5] Wertheim HF Melles DC Vos MC van Leeuwen W van Belkum A Verbrugh HA. The role of nasal carriage in Staphylococcus aureus infections. Lancet Infect Dis. (2005) 5:751–62. doi: 10.1016/S1473-3099(05)70295-4 16310147

[6] Young BC Votintseva AA Foster D Godwin H Miller RR Anson LW. Multi-site and nasal swabbing for carriage of Staphylococcus aureus : what does a single nose swab predict? J Hosp Infection. (2017) 96:232–7. doi: 10.1016/j.jhin.2017.01.015 PMC5490851 28246002

[7] Albrecht VS Limbago BM Moran GJ Krishnadasan A Gorwitz RJ McDougal LK. Staphylococcus aureus Colonization and Strain Type at Various Body Sites among Patients with a Closed Abscess and Uninfected Controls at U.S. Emergency Departments. J Clin Microbiol. (2015) 53:3478–84. doi: 10.1128/JCM.01371-15 PMC4609677 26292314

[8] Acton DS Tempelmans Plat-Sinnige MJ van Wamel W de Groot N van Belkum A. Intestinal carriage of Staphylococcus aureus: how does its frequency compare with that of nasal carriage and what is its clinical impact? Eur J Clin Microbiol Infect Dis. (2009) 28:115. doi: 10.1007/s10096-008-0602-7 18688664

[9] Peacock SJ Justice A Griffiths D de Silva GDI Kantzanou MN Crook D. Determinants of acquisition and carriage of staphylococcus aureus in infancy. J Clin Microbiol. (2003) 41:5718–25. doi: 10.1128/JCM.41.12.5718-5725.2003 PMC308978 14662966

[10] Key FM Khadka VD Romo-González C Blake KJ Deng L Lynn TC. On-person adaptive evolution of Staphylococcus aureus during treatment for atopic dermatitis. Cell Host Microbe. (2023) 31:593–603. doi: 10.1016/j.chom.2023.03.009 37054679 PMC10263175

[11] Hendriks A Mnich ME Clemente B Cruz AR Tavarini S Bagnoli F. Staphylococcus aureus-specific tissue-resident memory CD4+ T cells are abundant in healthy human skin. Front Immunol. (2021) 12:642711. doi: 10.3389/fimmu.2021.642711 33796109 PMC8008074

[12] Meyer TC Michalik S Holtfreter S Weiss S Friedrich N Völzke H. A comprehensive view on the human antibody repertoire against staphylococcus aureus antigens in the general population. Front Immunol. (2021) 12. doi: 10.3389/fimmu.2021.651619 PMC7987813 33777051

[13] Kolata JB Kühbandner I Link C Normann N Vu CH Steil L. The fall of a Dogma? Unexpected high T-cell memory response to staphylococcus aureus in humans. J Infect Diseases. (2015) 212:830–8. doi: 10.1093/infdis/jiv128 25737563

[14] Ferraro A Buonocore SM Auquier P Nicolas I Wallemacq H Boutriau D. Role and plasticity of Th1 and Th17 responses in immunity to *Staphylococcus aureus* . Hum Vaccin Immunother. (2019) 15:2980–92. doi: 10.1080/21645515.2019.1613126 PMC6930085 31149870

[15] Vella V Galgani I Polito L Arora AK Creech CB David MZ. Staphylococcus aureus Skin and Soft Tissue Infection Recurrence Rates in Outpatients: A Retrospective Database Study at 3 US Medical Centers. Clin Infect Dis. (2021) 73(5): e1045–53. doi: 10.1093/cid/ciaa1717 PMC8423503 33197926

[16] Tsang JS Schwartzberg PL Kotliarov Y Biancotto A Xie Z Germain RN. Global analyses of human immune variation reveal baseline predictors of postvaccination responses. Cell. (2014) 157:499–513. doi: 10.1016/j.cell.2014.03.031 24725414 PMC4139290

[17] Kim JH Skountzou I Compans R Jacob J. Original antigenic sin responses to influenza viruses. J Immunol. (2009) 183:3294–301. doi: 10.4049/jimmunol.0900398 PMC4460008 19648276

[18] Ebinger JE Fert-Bober J Printsev I Wu M Sun N Prostko JC. Antibody responses to the BNT162b2 mRNA vaccine in individuals previously infected with SARS-CoV-2. Nat Med. (2021) 27:981–4. doi: 10.1038/s41591-021-01325-6 PMC8205849 33795870

[19] Teymournejad O Li Z Beesetty P Yang C Montgomery CP. Toxin expression during Staphylococcus aureus infection imprints host immunity to inhibit vaccine efficacy. NPJ Vaccines. (2023) 8:3. doi: 10.1038/s41541-022-00598-3 36693884 PMC9873725

[20] Tsai CM Caldera JR Hajam IA Chiang AWT Tsai CH Li H. Non-protective immune imprint underlies failure of Staphylococcus aureus IsdB vaccine. Cell Host Microbe. (2022) 30:1163–1172.e6. doi: 10.1016/j.chom.2022.06.006 35803276 PMC9378590

[21] Lee B Olaniyi R Kwiecinski JM Wardenburg JB. Staphylococcus aureus toxin suppresses antigen-specific T cell responses. J Clin Invest. (2020) 130:1122–7. doi: 10.1172/JCI130728 PMC7269593 31873074

[22] Karauzum H Venkatasubramaniam A Adhikari RP Kort T Holtsberg FW Mukherjee I. IBT-V02: A multicomponent toxoid vaccine protects against primary and secondary skin infections caused by staphylococcus aureus. Front Immunol. (2021) 12. doi: 10.3389/fimmu.2021.624310 PMC7987673 33777005

[23] Bagnoli F Fontana MR Soldaini E Mishra RPN Fiaschi L Cartocci E. Vaccine composition formulated with a novel TLR7-dependent adjuvant induces high and broad protection against Staphylococcus aureus. Proc Natl Acad Sci U S A. (2015) 112:3680–5. doi: 10.1073/pnas.1424924112 PMC4378396 25775551

[24] Hoernes M Seger R Reichenbach J. Modern management of primary B-cell immunodeficiencies. Pediatr Allergy Immunol. (2011) 22:758–69. doi: 10.1111/j.1399-3038.2011.01236.x 22122788

[25] Dhalla F Misbah SA. Secondary antibody deficiencies. Curr Opin Allergy Clin Immunol. (2015) 15:505–13. doi: 10.1097/ACI.0000000000000215 26406183

[26] Armentrout E Liu G Martins G. T Cell Immunity and the Quest for Protective Vaccines against Staphylococcus aureus Infection. Microorganisms. (2020) 8:1936. doi: 10.3390/microorganisms8121936 33291260 PMC7762175

[27] Montgomery CP Daniels M Zhao F Alegre ML Chong AS Daum RS. Protective immunity against recurrent Staphylococcus aureus skin infection requires antibody and interleukin-17A. Infect Immun. (2014) 82:2125–34. doi: 10.1128/IAI.01491-14 PMC3993461 24614654

[28] Brown AF Murphy AG Lalor SJ Leech JM O’Keeffe KM Mac Aogáin M. Memory th1 cells are protective in invasive staphylococcus aureus infection. PloS Pathog. (2015) 11. doi: 10.1371/journal.ppat.1005226 PMC4634925 26539822

[29] Mancini F Monaci E Lofano G Torre A Bacconi M Tavarini S. One dose of staphylococcus aureus 4C-staph vaccine formulated with a novel TLR7-dependent adjuvant rapidly protects mice through antibodies, effector CD4+ T Cells, and IL-17A. PloS One. (2016) 11. doi: 10.1371/journal.pone.0147767 PMC4727907 26812180

[30] Crum-Cianflone NF Burgi AA Hale BR. Increasing rates of community-acquired methicillin-resistant Staphylococcus aureus infections among HIV-infected persons. Int J STD AIDS. (2007) 18:521–6. doi: 10.1258/095646207781439702 17686212

[31] Minegishi Y Saito M Nagasawa M Takada H Hara T Tsuchiya S. Molecular explanation for the contradiction between systemic Th17 defect and localized bacterial infection in hyper-IgE syndrome. J Exp Med. (2009) 206:1291–301. doi: 10.1084/jem.20082767 PMC2715068 19487419

[32] Tangye SG Puel A. The th17/IL-17 axis and host defense against fungal infections. J Allergy Clin Immunol Pract. (2023) 11:1624–34. doi: 10.1016/j.jaip.2023.04.015 PMC12664666 37116791

[33] Braverman J Monk IR Ge C Westall GP Stinear TP Wakim LM. Staphylococcus aureus specific lung resident memory CD4+ Th1 cells attenuate the severity of influenza virus induced secondary bacterial pneumonia. Mucosal Immunol. (2022) 15:783–96. doi: 10.1038/s41385-022-00529-4 PMC9148937 35637249

[34] DuPage M Bluestone JA. Harnessing the plasticity of CD4+ T cells to treat immune-mediated disease. Nat Rev Immunol. (2016) 16:149–63. doi: 10.1038/nri.2015.18 26875830

[35] Singh SP Parween F Edara N Zhang HH Chen J Otaizo-Carrasquero F. Human CCR6+ Th cells show both an extended stable gradient of th17 activity and imprinted plasticity. J Immunol. (2023) 210:1700–16. doi: 10.4049/jimmunol.2200874 PMC10463241 37093875

[36] Muraro MJ Dharmadhikari G Grün D Groen N Dielen T Jansen E. A single-cell transcriptome atlas of the human pancreas. Cell Syst. (2016) 3:385–94. doi: 10.1016/j.cels.2016.09.002 PMC5092539 27693023

[37] Wolock SL Lopez R Klein AM. Scrublet: computational identification of cell doublets in single-cell transcriptomic data. Cell Syst. (2019) 8:281–91. doi: 10.1016/j.cels.2018.11.005 PMC6625319 30954476

[38] Polański K Young MD Miao Z Meyer KB Teichmann SA Park JE. BBKNN: fast batch alignment of single cell transcriptomes. Bioinformatics. (2020) 36:964–5. doi: 10.1093/bioinformatics/btz625 PMC9883685 31400197

[39] Traag VA Waltman L van Eck NJ. From Louvain to Leiden: guaranteeing well-connected communities. Sci Rep. (2019) 9:5233. doi: 10.1038/s41598-019-41695-z 30914743 PMC6435756

[40] Fang Z Liu X Peltz G. GSEApy: a comprehensive package for performing gene set enrichment analysis in Python. Bioinformatics. (2023) 39. doi: 10.1093/bioinformatics/btac757 PMC9805564 36426870

[41] Faure L Soldatov R Kharchenko PV Adameyko I. scFates: a scalable python package for advanced pseudotime and bifurcation analysis from single-cell data. Bioinformatics. (2023) 39. doi: 10.1093/bioinformatics/btac746 PMC9805561 36394263

[42] Leuzzi R Bodini M Thomsen IP Soldaini E Bartolini E Muzzi A. Dissecting the human response to staphylococcus aureus systemic infections. Front Immunol. (2021) 12. doi: 10.3389/fimmu.2021.749432 PMC8607524 34819932

[43] Soneson C Robinson MD. Bias, robustness and scalability in single-cell differential expression analysis. Nat Methods. (2018) 15:255–61. doi: 10.1038/nmeth.4612 29481549

[44] Poloni C Schonhofer C Ivison S Levings MK Steiner TS Cook L. T-cell activation–induced marker assays in health and disease. Immunol Cell Biol. (2023) 101:491–503. doi: 10.1111/imcb.v101.6 36825901 PMC10952637

[45] Grifoni A Weiskopf D Ramirez SI Mateus J Dan JM Moderbacher CR. Targets of T cell responses to SARS-coV-2 coronavirus in humans with COVID-19 disease and unexposed individuals. Cell. (2020) 181:1489–501. doi: 10.1016/j.cell.2020.05.015 PMC7237901 32473127

[46] Reiss S Baxter AE Cirelli KM Dan JM Morou A Daigneault A. Comparative analysis of activation induced marker (AIM) assays for sensitive identification of antigen-specific CD4 T cells. PloS One. (2017) 12:e0186998. doi: 10.1371/journal.pone.0186998 29065175 PMC5655442

[47] Strobl J Gail LM Kleissl L Pandey RV Smejkal V Huber J. Human resident memory T cells exit the skin and mediate systemic Th2-driven inflammation. J Exp Med. (2021) 218. doi: 10.1084/jem.20210417 PMC8563284 34643646

[48] de Almeida GP Lichtner P Eckstein G Brinkschmidt T Chu CF Sun S. Human skin-resident host T cells can persist long term after allogeneic stem cell transplantation and maintain recirculation potential. Sci Immunol. (2022) 7. doi: 10.1126/sciimmunol.abe2634 35089814

[49] Klicznik MM Morawski PA Höllbacher B Varkhande SR Motley SJ Kuri-Cervantes L. Human CD4+CD103+ cutaneous resident memory T cells are found in the circulation of healthy individuals. Sci Immunol. (2019) 4. doi: 10.1126/sciimmunol.aav8995 PMC7057121 31278120

[50] Poon MML Caron DP Wang Z Wells SB Chen D Meng W. Tissue adaptation and clonal segregation of human memory T cells in barrier sites. Nat Immunol. (2023) 24:309–19. doi: 10.1038/s41590-022-01395-9 PMC10063339 36658238

[51] Szabo PA Levitin HM Miron M Snyder ME Senda T Yuan J. Single-cell transcriptomics of human T cells reveals tissue and activation signatures in health and disease. Nat Commun. (2019) 10:4706. doi: 10.1038/s41467-019-12464-3 31624246 PMC6797728

[52] Wang C Kang SG Lee J Sun Z Kim CH. The roles of CCR6 in migration of Th17 cells and regulation of effector T-cell balance in the gut. Mucosal Immunol. (2009) 2:173–83. doi: 10.1038/mi.2008.84 PMC2709747 19129757

[53] Liu W Putnam AL Xu-yu Z Szot GL Lee MR Zhu S. CD127 expression inversely correlates with FoxP3 and suppressive function of human CD4+ T reg cells. J Exp Med. (2006) 203:1701–11. doi: 10.1084/jem.20060772 PMC2118339 16818678

[54] Joller N Lozano E Burkett PR Patel B Xiao S Zhu C. Treg cells expressing the coinhibitory molecule TIGIT selectively inhibit proinflammatory th1 and th17 cell responses. Immunity. (2014) 40:569–81. doi: 10.1016/j.immuni.2014.02.012 PMC4070748 24745333

[55] Joller N Anderson AC Kuchroo VK. LAG-3, TIM-3, and TIGIT: Distinct functions in immune regulation. Immunity. (2024) 57:206–22. doi: 10.1016/j.immuni.2024.01.010 PMC10919259 38354701

[56] Cho JS Pietras EM Garcia NC Ramos RI Farzam DM Monroe HR. IL-17 is essential for host defense against cutaneous Staphylococcus aureus infection in mice. J Clin Invest. (2010) 120:1762–73. doi: 10.1172/JCI40891 PMC2860944 20364087

[57] Fries A Saidoune F Kuonen F Dupanloup I Fournier N Guerra de Souza AC. Differentiation of IL-26+ TH17 intermediates into IL-17A producers via epithelial crosstalk in psoriasis. Nat Commun. (2023) 14:3878. doi: 10.1038/s41467-023-39484-4 37391412 PMC10313793

[58] Dambacher J Beigel F Zitzmann K De Toni EN Goke B Diepolder HM. The role of the novel Th17 cytokine IL-26 in intestinal inflammation. Gut. (2009) 58:1207–17. doi: 10.1136/gut.2007.130112 18483078

[59] Larochette V Miot C Poli C Beaumont E Roingeard P Fickenscher H. IL-26, a cytokine with roles in extracellular DNA-induced inflammation and microbial defense. Front Immunol. (2019) 10. doi: 10.3389/fimmu.2019.00204 PMC6379347 30809226

[60] Woetmann A Alhede M Dabelsteen S Bjarnsholt T Rybtke M Nastasi C. Interleukin-26 (IL-26) is a novel anti-microbial peptide produced by T cells in response to staphylococcal enterotoxin. Oncotarget. (2018) 9:19481–9. doi: 10.18632/oncotarget.24603 PMC5929403 29731960

[61] Agak GW Mouton A Teles RMB Weston T Morselli M Andrade PR. Extracellular traps released by antimicrobial TH17 cells contribute to host defense. J Clin Invest. (2021) 131. doi: 10.1172/JCI141594 PMC7810473 33211671

[62] Luna BM Nielsen TB Cheng B Pantapalangkoor P Yan J Boyle-Vavra S. Vaccines targeting Staphylococcus aureus skin and bloodstream infections require different composition. PloS One. (2019) 14. doi: 10.1371/journal.pone.0217439 PMC6557488 31181086

[63] Beesetty P Si Y Li Z Yang C Zhao F Chong AS. Tissue specificity drives protective immunity against Staphylococcus aureus infection. Front Immunol. (2022) 13. doi: 10.3389/fimmu.2022.795792 PMC9380724 35983063

[64] Bacher P Heinrich F Stervbo U Nienen M Vahldieck M Iwert C. Regulatory T Cell Specificity Directs Tolerance versus Allergy against Aeroantigens in Humans. Cell. (2016) 167:1067–1078.e16. doi: 10.1016/j.cell.2016.09.050 27773482

[65] Lundell A Adlerberth I Lindberg E Karlsson H Ekberg S Åberg N. Increased levels of circulating soluble CD14 but not CD83 in infants are associated with early intestinal colonization with *Staphylococcus aureus* . Clin Exp Allergy. (2007) 37:62–71. doi: 10.1111/j.1365-2222.2006.02625.x 17210043

[66] Rabe H Nordström I Andersson K Lundell A Rudin A. Staphylococcus aureus convert neonatal conventional CD4+ T cells into FOXP3+ CD25+ CD127low T cells via the PD-1/PD-L1 axis. Immunology. (2014) 141:467–81. doi: 10.1111/imm.2014.141.issue-3 PMC3930383 24708420

[67] Leech JM Dhariwala MO Lowe MM Chu K Merana GR Cornuot C. Toxin-triggered interleukin-1 receptor signaling enables early-life discrimination of pathogenic versus commensal skin bacteria. Cell Host Microbe. (2019) 26:795–809.e5. doi: 10.1016/j.chom.2019.10.007 31784259 PMC6989301

[68] Yu X Harden K C Gonzalez L Francesco M Chiang E Irving B. The surface protein TIGIT suppresses T cell activation by promoting the generation of mature immunoregulatory dendritic cells. Nat Immunol. (2009) 10:48–57. doi: 10.1038/ni.1674 19011627

[69] Joller N Hafler JP Brynedal B Kassam N Spoerl S Levin SD. Cutting edge: TIGIT has T cell-intrinsic inhibitory functions. J Immunol. (2011) 186:1338–42. doi: 10.4049/jimmunol.1003081 PMC3128994 21199897

[70] Worboys JD Vowell KN Hare RK Ambrose AR Bertuzzi M Conner MA. TIGIT can inhibit T cell activation via ligation-induced nanoclusters, independent of CD226 co-stimulation. Nat Commun. (2023) 14:5016. doi: 10.1038/s41467-023-40755-3 37596248 PMC10439114

[71] Acosta-Rodriguez EV Napolitani G Lanzavecchia A Sallusto F. Interleukins 1β and 6 but not transforming growth factor-β are essential for the differentiation of interleukin 17–producing human T helper cells. Nat Immunol. (2007) 8:942–9. doi: 10.1038/ni1496 17676045

[72] Lasigliè D Traggiai E Federici S Alessio M Buoncompagni A Accogli A. Role of IL-1 beta in the development of human TH17 cells: lesson from NLPR3 mutated patients. PloS One. (2011) 6:e20014. doi: 10.1371/journal.pone.0020014 21637346 PMC3102666

[73] Zielinski CE Mele F Aschenbrenner D Jarrossay D Ronchi F Gattorno M. Pathogen-induced human TH17 cells produce IFN-γ or IL-10 and are regulated by IL-1β. Nature. (2012) 484:514–8. doi: 10.1038/nature10957 22466287

[74] Zhao J Chen Y Ding ZY Liu JY. Safety and efficacy of therapeutic cancer vaccines alone or in combination with immune checkpoint inhibitors in cancer treatment. Front Pharmacol. (2019) 10. doi: 10.3389/fphar.2019.01184 PMC6798079 31680963

[75] Karyampudi L Lamichhane P Scheid AD Kalli KR Shreeder B Krempski JW. Accumulation of memory precursor CD8 T cells in regressing tumors following combination therapy with vaccine and anti-PD-1 antibody. Cancer Res. (2014) 74:2974–85. doi: 10.1158/0008-5472.CAN-13-2564 PMC4313351 24728077

[76] Läubli H Balmelli C Kaufmann L Stanczak M Syedbasha M Vogt D. Influenza vaccination of cancer patients during PD-1 blockade induces serological protection but may raise the risk for immune-related adverse events. J Immunother Cancer. (2018) 6:40. doi: 10.1186/s40425-018-0353-7 29789020 PMC5964701

[77] Hultgren OH Svensson L Tarkowski A. Critical role of signaling through IL-1 receptor for development of arthritis and sepsis during staphylococcus aureus infection. J Immunol. (2002) 168:5207–12. doi: 10.4049/jimmunol.168.10.5207 11994477

[78] Kielian T Bearden ED Baldwin AC Esen N. IL-1 and TNF-α Play a pivotal role in the host immune response in a mouse model of *staphylococcus aureus* -induced experimental brain abscess. J Neuropathol Exp Neurol. (2004) 63:381–96. doi: 10.1093/jnen/63.4.381 15099027

[79] Verdrengh M Thomas JA Hultgren OH. IL-1 receptor-associated kinase 1 mediates protection against Staphylococcus aureus infection. Microbes Infect. (2004) 6:1268–72. doi: 10.1016/j.micinf.2004.08.009 15555532

[80] Miller LS O’Connell RM Gutierrez MA Pietras EM Shahangian A Gross CE. MyD88 Mediates Neutrophil Recruitment Initiated by IL-1R but Not TLR2 Activation in Immunity against Staphylococcus aureus. Immunity. (2006) 24:79–91. doi: 10.1016/j.immuni.2005.11.011 16413925

[81] Rose WE Eickhoff JC Shukla SK Pantrangi M Rooijakkers S Cosgrove SE. Elevated Serum Interleukin-10 at Time of Hospital Admission Is Predictive of Mortality in Patients With Staphylococcus aureus Bacteremia. J Infect Dis. (2012) 206:1604–11. doi: 10.1093/infdis/jis552 PMC6281403 22966128

[82] de Lima MHF MaChado CC Nascimento DC Silva CMS Toller-Kawahisa JE Rodrigues TS. The TIGIT+ T regulatory cells subset associates with nosocomial infection and fatal outcome in COVID-19 patients under mechanical ventilation. Sci Rep. (2023) 13:13599. doi: 10.1038/s41598-023-39924-7 37604833 PMC10442317

[83] Hu ZI Link VM Lima-Junior DS Delaleu J Bouladoux N Han SJ. Immune checkpoint inhibitors unleash pathogenic immune responses against the microbiota. Proc Natl Acad Sci. (2022) 119. doi: 10.1073/pnas.2200348119 PMC9245641 35727974

