# Supplementary material for: Staphylococcus aureus-specific TIGIT+ Treg are present in the blood of healthy subjects – a hurdle for vaccination?
Source: Front Immunol. 2025 Feb 4;15:1500696. doi: 10.3389/fimmu.2024.1500696 (PMC11840346; doi:10.3389/fimmu.2024.1500696)
Supplement: Supplementary file 1 [file DataSheet1.docx]

**Supplementary material**

**Supplementary Table S1. Table of antibodies used in flow cytometry and respective fluorochrome and clone.**

| Cell target | Fluorochrome | Clone | Dilution | Catalogue number | Supplier |
| --- | --- | --- | --- | --- | --- |
| Cell viability | Near-IR | LIVE/DEAD™ Fixable Dead Cell Stain Kit | 1000 | L10119 | Invitrogen |
| Cell viability | 440UV | BD Horizon™ Fixable Viability Stain | 1000 | 566332 | BD |
| CD3 | APC-H7 | SK7 | 50 | 560176 | BD |
| CD3 | PE-CF594 | UCHT1 | 200 | 562280 | BD |
| CD4 | BUV395 | RPA-T4 | 50 | 564724 | BD |
| CD4 | BV421 | RPA-T4 | 20 | 562424 | BD |
| CD8 | BV605 | SK1 | 100 | 564116 | BD |
| CD8 | BUV805 | RPA-T8 | 200 | 749366 | BD |
| OX40 | PE-Cy7 | Ber-ACT35 | 320 | 350012 | Biolegend |
| OX40 | BB660 | Ber-ACT35 | 25 | CUSTOM | BD |
| CD137 | APC | 4B4-1 | 60 | 309810 | Biolegend |
| CD137 | BUV661 | 4B4-1 | 100 | 741642 | BD |
| CLA | BV605 | HECA-452 | 40 | 563960 | BD |
| CLA | BB755 | HECA-452 | 80 | 624391 | BD |
| CD25 | PE-CF594 | BC96 | 100 | 567489 | BD |
| CD25 | PE-CF594 | M-A251 | 100 | 562403 | BD |
| CD40L | BV480 | 24-31 | 30 | 752858 | BD |
| CD127 | BB700 | HIL-7R-M21 | 400 | 566398 | BD |
| FOXP3 | BB700 | 236A/E7 | 40 | 566526 | BD |
| CTLA-4 | PE-Cy™5 | BNI3 | 20 | 555854 | BD |
| TIGIT | PE | TgMab-2 | 25 | 568672 | BD |
| IFN-γ | BV570 | B27 | 60 | 624298 | BD |
| IL-17A | BV786 | N49-653 | 25 | 563745 | BD |
| IL-26 | PE | 510414 | 20 | MA5-23678 | Invitrogen |


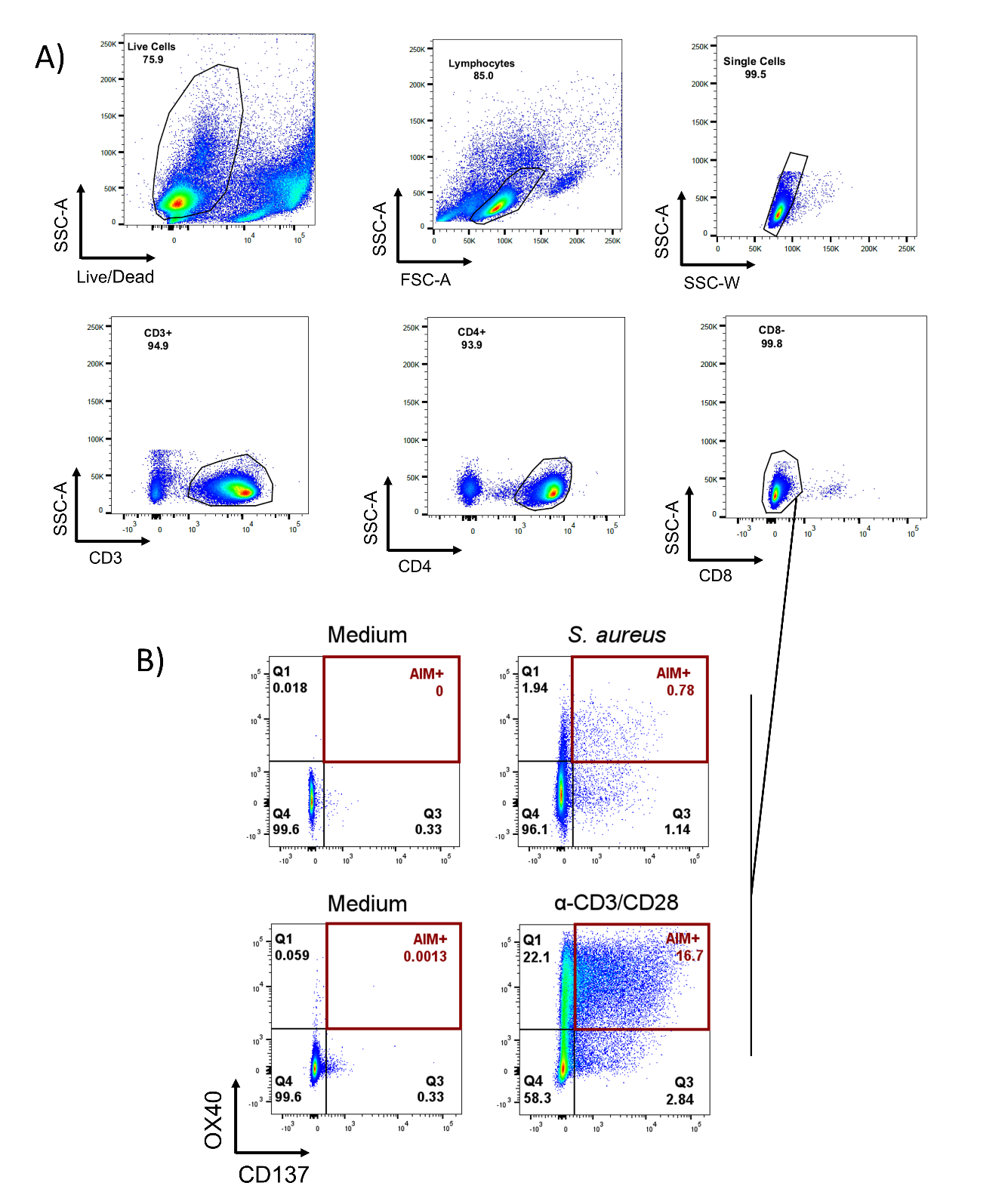


**Supplementary Figure S1.** **Gating strategy used to identify AIM^+^ CD4^+^ T cells**

PBMCs isolated from healthy donors were enriched via negative selection into CD4^+^ and CD4^-^ fractions. CD4^-^ cells were irradiated and both fractions were re-mixed at a ratio of 1:1. Cells were then stimulated for 24 h with HK *S. aureus* or α-CD3/CD28 or left untreated. (**A**) “CD4^+^ T cells” were identified by gating on live, morphology of lymphocytes, singlets, CD3^+^, CD4^+^, CD8^-^ cells. These cells were then assayed for the expression of 2 activation markers: OX40 and CD137 with double positive cells being denoted as AIM^+^. (**B**) Representative gating and expression of both activation markers.


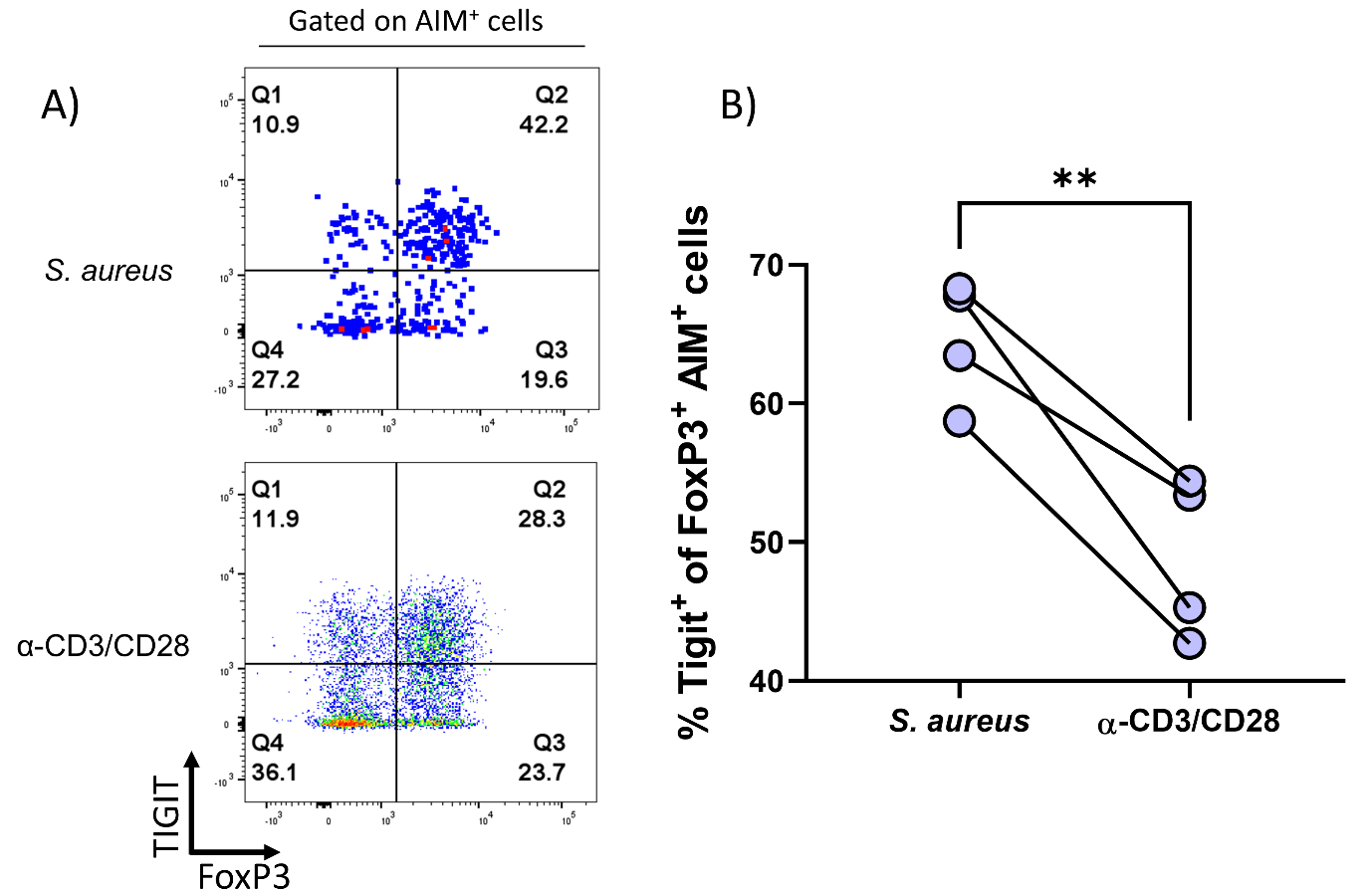


**Supplementary Figure S2. TIGIT^+^ Treg are enriched in S. aureus-specific as compared to polyclonally-activated CD4^+^ T cells**

PBMCs isolated from healthy donors were sorted via negative selection into CD4^+^ and CD4^-^ fractions. CD4^-^ cells were irradiated and both fractions were re-mixed at a ratio of 1:1. Cells were then stimulated for 24 h with HK *S. aureus* or α-CD3/CD28. TIGIT expression on *S. aureus*-specific Treg was measured via flow cytometry by first gating on AIM^+^ (OX40^+^ CD137^+^) CD4^+^ T cells expressing the Treg marker FoxP3. (**A**) Representative flow cytometry staining of FoxP3 and TIGIT expression on AIM^+^ cells. (**B**) The percentage of *S. aureus*-specific Treg expressing TIGIT was compared to polyclonally-activated Treg for 4 donors. Statistical analysis was performed using a paired t-test. ***P* ≤ 0.01.


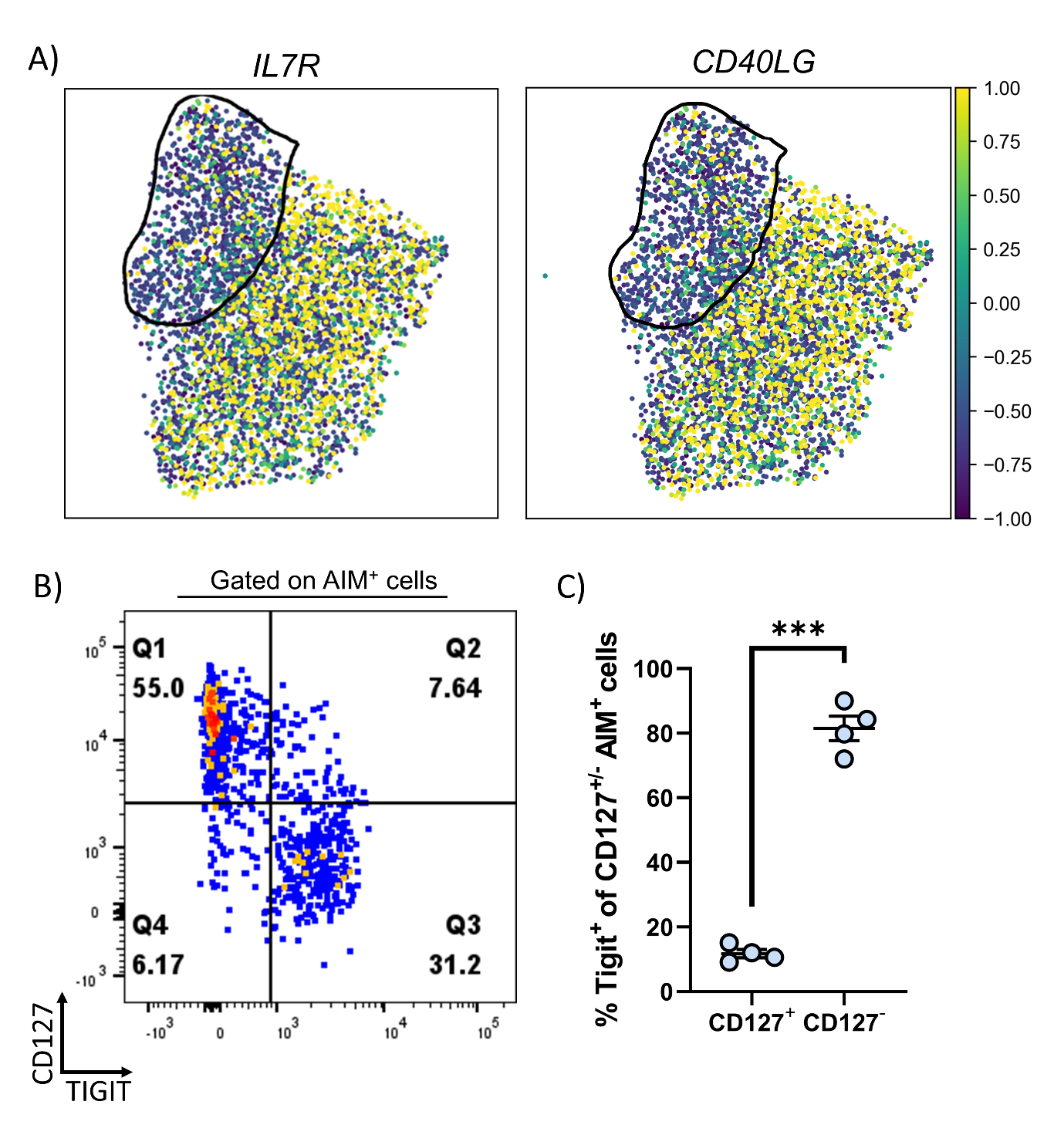


**Supplementary Figure S3. S. aureus-specific Treg express neither CD127 nor CD40L at the transcriptional and protein level**

PBMCs isolated from 6 healthy donors were sorted via negative selection into CD4^+^ and CD4^-^ fractions. CD4^-^ cells were irradiated and both fractions were re-mixed at a ratio of 1:1. Cells were then stimulated for 24 h with HK-*S. aureus* or α-CD3/CD28. Cells co-expressing the activation markers CD137 and OX40 were analysed via scRNA-seq. (**A)** Relative expression of the genes *IL7R* (encoding CD127) and *CD40LG* (encoding CD40L) was measured. The Treg cluster is circled in black. (**B**) Expression of CD127 and TIGIT on AIM^+^ cells was measured via flow cytometry. (**C**) Graphical representation of TIGIT expression as determined via FACS on CD127^+^ and CD127^-^ *S. aureus*-specific AIM^+^ cells (*n* = 4 donors). Statistical analysis was performed using a paired t-test. ****P* ≤ 0.001.
